# *Enterococcus durans* with mosquito larvicidal toxicity against *Culex quinquefasciatus*, elucidated using a Proteomic and Metabolomic approach

**DOI:** 10.1038/s41598-020-61245-2

**Published:** 2020-03-16

**Authors:** Domnic Colvin, Vishnu Dhuri, Hirday Verma, Rama Lokhande, Avinash Kale

**Affiliations:** 1 School of Chemical Sciences, UM-DAE Centre for Excellence in Basic Sciences, University of Mumbai, Kalina, Santacruz –East, Mumbai, 400098 India; 2 Jaipur National University, Agra - Jaipur Rd, Near New RTO Office, Jagatpura, Jaipur, Rajasthan 302017 India

**Keywords:** Applied microbiology, Metabolomics, Proteomics, Sequencing, Metabolomics, Natural products, Proteomics, Mass spectrometry, Electron microscopy, NMR spectroscopy

## Abstract

Various bacteria from the *Bacillus* species have been used as pesticides against mosquito larvae for more than a decade. The prolonged use of these bacterial species by little alteration within their genome, using various permutations and combinations of mosquito-cidal toxins, has proven unsuccessful in controlling the mosquito population. In our current study we report *Enterococcus sp*. to be exhibiting similar kind of mosquito-cidal toxins alike those which are present in the mainly used *Bacillus* strains. Three *Enterococcus* species were isolated on a rich media selective for gram- positive bacteria from the mid-gut of dead mosquito larvae which were collected from the wild locations within and around the city of Mumbai, India. Their surface morphologies were studied by Scanning Electron Microscopy (SEM) and their identity was confirmed using the standard 16S rRNA sequencing method. Upon performing several repetitive toxicity assays of these three strains on the laboratory cultured third instar stage of *Culex quinquefasciatus* larvae, showed differential toxicities from a minimum of 20% (LC_50_: 59.6 CFU/ml), intermediate 35% (LC_50_: 48.4 CFU/ml) and a maximum of 60% (LC_50_: 35.7 CFU/ml). To justify the data in all the three similar strains of *Enterococcus durans*, we followed the differential proteomics using LCMS 6540 UHD Accurate Mass QTOF and differential metabolomics approach using both LCMS 6540 UHD Accurate Mass QTOF and ^1^H-NMR. The presence and significance of the obtained toxins were studied to elucidate the plausible reason for showing differential toxicities. This work helped in identifying *Enterococcus durans* as a new, potential and alternative strain to the *Bacillus* species in terms of mosquito larvicidal toxicity against *Culex quinquefasciatus*.

## Introduction

Mosquito transmitted diseases such as malaria, dengue, yellow fever, Japanese encephalitis and filariasis is responsible for the high number of human mortalities across the globe. There have been some successes in controlling the death tolls by vaccines and drug treatment, but most of the current measures for limiting these diseases focus on vector control. The use of bed-nets, habitat modifications, sterile-male mosquito releases and mosquito-cidal insecticides were tried historically to control vector population. But over the past 15 years, substantial progress has been made in developing alternative biocontrol methods through bacterial symbionts for biological control of mosquitoes^1^. Microbial larvicides like *Bacillus thuringiensis* var. *israelensis* (*Bt*) and *Bacillus sphaericus* (*Bs*) have been found to be effective and used extensively against mosquitoes with being safe to non-target organisms^2^. The mosquito-cidal endotoxins of *Bt* and *Bs* have various environmental benefits including safety for humans and other organisms, reduction of pesticides residue in the aquatic environment, increased activity of most other natural enemies and increased biodiversity in aquatic ecosystem. The advent of recombinant DNA technology which enables to manipulate and recombine genes have improved microbial larvicides making them more potent as many synthetic insecticides for vector control^3^.

Unfortunately, studies have revealed that both these strains are now at a high risk of resistance development in mosquito population^4,5^. It has also been suggested that mixture of endotoxin components from *Bs* and *Bt*, which react synergistically, will offer longer lasting and more effective mosquito control than the individual strains alone^5^. But recently it was also found that cross resistance is wide-spread among mosquito-cidal *Bt* Cry toxins and the mechanism of Cry resistance in mosquitoes are not known. Many field cases from a number of locations worldwide have reported that *Bs* is also at a higher risk of resistance due to its single site action^4^. Traditional resistance management strategies of using rotations and mixtures of *Bt* and *Bs* by genetic engineering needs to be substituted with promising new strategies to increase the toxin complexity targeted towards mosquito larvae, to enhance the host range of the mosquito control product and to avoid evolution of insecticide resistance.

Thus, screening of new bacterial species exhibiting similar mosquito-cidal toxins as that of *Bt* and *Bs* are required to counter the evolution of resistance in the current target mosquito population. An attempt was made for the same by isolating gram- positive bacteria from the mid-gut microflora of dead *Culex quinquefasciatus* (*C. quinquefasciatus*) mosquito larvae which were collected from different wild locations from Mumbai, India. These isolated bacteria were tested for their toxicity against the third instar stage of the lab cultured *C. quinquefasciatus* mosquito larvae. The three bacterial strains which showed differential toxicities were identified using 16S rRNA sequencing. All the three were identified as *Enterococcus durans* (*E. durans*) and their surface morphology was identified using Scanning Electron Microscopy. To the best of our knowledge for the first time we report the mosquito larvicidal activity of *E. durans* ranging from 20% to 60% per CFU (colony forming unit) and the rational of their differential toxicities is reported using proteomics and metabolomics studies.

The emergence of high through-put proteomics and metabolomics using LCMS helps in generation of large scale data set which can be used for the identification of toxicity markers of various mosquito larvicides. In our current study, we made use of a novel combined approach of proteomics and metabolomics on our identified *E. durans* strains for elucidating the potential mosquito larvicidal components in them. The 1H NMR spectra of the metabolites isolated from the three strains of this bacteria were also studied to illustrate, compare and strengthen our results of the differential toxicities obtained from our toxicity assay. Additionally, we also carried out *in vitro* cytotoxicity analysis of the proteins and metabolites from these three strains on the NCTC clone 929 (L cell, L-929, derivative of strain L- mouse lung fibroblast) cell lines to further ensure their non-toxic characteristics against the non-target eukaryotic organisms in the environment.

## Results

### Mosquito larvicidal activity

The bacteria which were screened on the gram-positive selective media, were tested for their toxicity against the third instar stage of *C. quinquefasciatus* mosquito larvae. The toxicity assay was repeated five times to obtain a statistically relevant data for these three strains; namely S1, S2 and S3 which showed toxicity of 60%, 35% and 20% respectively. The toxicity data for their mosquito larvicidal activity was subjected to probit analysis and their LC_50_ values were calculated. The details of the entire statistical analysis along with the equation of probit analysis and the goodness-of-fit test used are given in the Supplementary datasheet 1. The larvicidal activity in terms of LC_50_ values against *C. quinquefasciatus* was 35.67 CFU/ml for Strain S1, 48.43 CFU/ml for Strain S2 and 59.63 CFU/ml for Strain S3 as shown in Table 1. (CFU/ ml – colony forming units per milliliter). These values on conversion corresponds to 35.67 × 10^−3^ ng/ml for S1, 48.43 × 10^−3^ ng/ml for S2 and 59.63 × 10^−3^ ng/ml for S3 (If bacterial mass of 1 bacterium is considered to be 1 pico gram)^6,7^.

**Table 1:** Larvicidal toxicity of *Enterococcus durans* against third instar stage of *Culex quinquefasciatus* mosquito larvae.

Strain No.	% Toxicity	Larva (CFU/ml)	95% Fiducial Limit		Χ^2^ (d*f* = 3)
		LC_50_	LFL	UFL	
S1	60%	35.7	25.5	59.5	2.6
S2	35%	48.4	33.9	119.3	1.75
S3	20%	59.6	39.7	573.9	2.6

Control-Nil mortality, LFL = Lower Fiducial Limit, UFL = Upper Fiducial Limit, Χ^2^ –Chi-square value, df - degrees of freedom, CFU – colony forming units.

### Identification of the bacteria which are toxic against *C. quinquefasciatus*

These three bacterial strains showing differential toxicities were identified using 16S rRNA sequencing. Based on their morphological tests it was found that all are gram-positive cocci (spherical shaped). They were sequenced and confirmed by using five universal primers at NCMR, Pune. The 16S rRNA sequencing results of these bacterial species were compared to the NCBI reference database. All the three bacteria were identified as *E. durans* with an identity ranging from 96% to 98%. The maximum toxic (60%) strain S1 had a maximum score of 1633 and 98% query coverage with an identity of 96%. The max score of 1485 was observed in the medial toxic strain S2 showing 35% toxicity with 96% identity and 94% of query coverage. The least toxic strain S3 had only 20% toxicity which showed a max score of 1707 and 98% identity with query coverage of 94%. The entire details of the sequencing results are shown in Table 2.

**Table 2:** Identification of the *Enterococcus species* using 16S rRNA sequencing and comparing against NCBI database.

		Toxicity	Description	Strain	Max score	Total score	Query cover	E- value	Identity
Strain no. 3	S3	20%	*Enterococcus durans*	JCM 8725	1707	2853	94%	0.0	98%
			*Enterococcus durans*	NBRC 100479	1707	2857	94%	0.0	98%
			*Enterococcus durans*	98D	1707	2892	95%	0.0	98%
Strain no. 2	S2	35%	*Enterococcus durans*	JCM 8725	1485	2753	94%	0.0	96%
			*Enterococcus durans*	NBRC 100479	1485	2757	94%	0.0	96%
			*Enterococcus durans*	98D	1485	2757	94%	0.0	96%
Strain no.1	S1	60%	*Enterococcus durans*	JCM 8725	1633	2886	98%	0.0	96%
			*Enterococcus durans*	NBRC 100479	1633	2890	98%	0.0	96%
			*Enterococcus durans*	98D	1633	2919	99%	0.0	96%

Enterococcus durans strain JCM8725. NCBI Accession number: NR_113257.

*Enterococcus durans* strain NBRC 100479. NCBI Accession number: AB681177.

*Enterococcus durans* strain 98D. NCBI Accession number: NR_036922.

### SEM imaging of Bacteria

**Figure 1:**
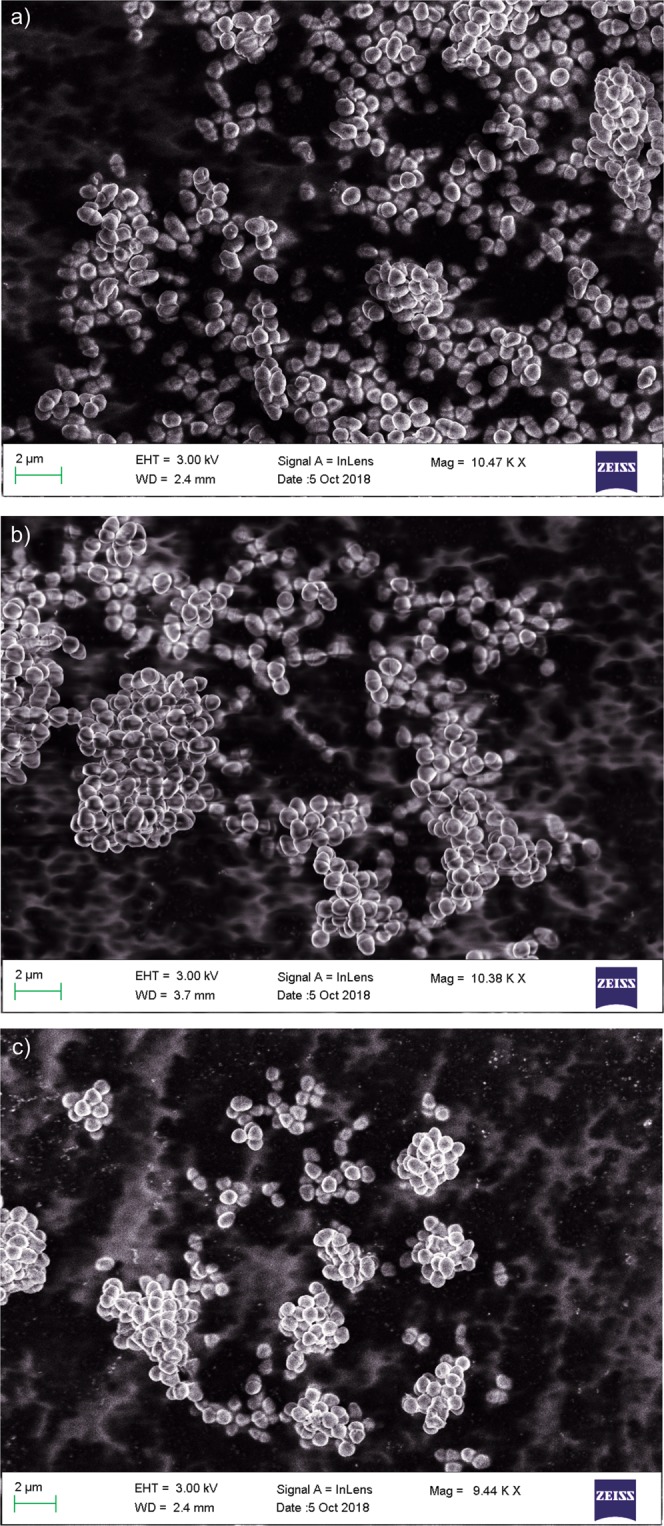
The SEM images of identified bacteria: (**a**) strain 1 (S1); (**b**) strain 2 (S2); and (**c**) strain 3 (S3) using ZEISS Ultra-field Emission SEM.

The three identified *E. durans* were imaged using Scanning electron microscopy to study their structural morphology. The results are shown in the Fig. 1a–c for the strain S1, S2 and S3 respectively. The basic morphology of *Enterococcus species* as per literature 10.1371/journal.pone.0118800.g005^8^ resembles the SEM images of our study. Also, our SEM images showed the presence of cocci shaped spherical cells joined together in duplicates which are the basic characteristics of *E. durans*^9^. This compliments our 16S rRNA results of identification by further ensuring that all the three strains are *E. durans*.

### Proteins and MS data acquisition

The quantitative analysis of the extracted proteins was carried out by Bradford’s assay and the concentration for Strain S1, S2 and S3 were found to be 15.45 µg/µl, 15.22 µg/µl and 19.96 µg/µl respectively. These samples were further subjected to in-solution trypsin digestion followed by LC-MS/MS analysis. (µg/µl: microgram per microliter).

**Figure 2:**
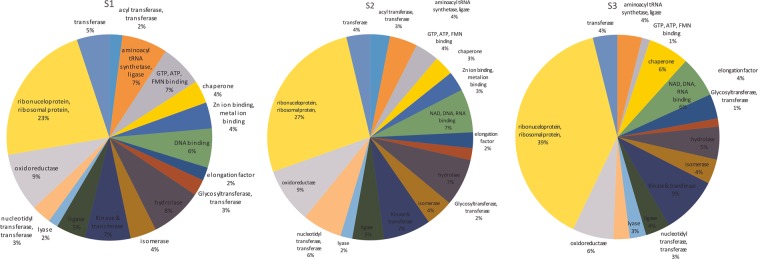
Distribution of the housekeeping proteins identified searching LCMS data against the available NCBI database for three strains (S1; S2; and S3) of *E. durans*.

The protein sequencing was carried out on nano-LCMS by using 6540 UHD Accurate Mass QTOF (CAMS, Venture Center, NCL Pune). Peptide sequencing of these three strains S1, S2 and S3 resulted in the acquisition of 10,488, 9815 and 10,741 MS/MS spectra, respectively. More details on the obtained peaks can be found in Supplementary data 2, 3 and 4. These spectral peaks were compared with the UniProt protein databases of *E. durans* strain NCTC8130 (Proteome ID: UP000254113) to search for peptides matching mosquito-cidal toxins or proteins which are toxic. The UniProt database search results of all the three strains S1, S2 and S3 are provided in the Supplementary data Sheet 2. All the hits corresponding to the basic proteins were graphically compared within all the three strains showing no substantial differences in their proteome (Fig. 2). Therefore the high resolution MS/MS spectra was further searched against the customized toxin database (Cry, Bin, VIP and Mtx) incorporated with the Morpheus software suite^10^.

### MS data analysis

#### Proteins identified in customized database

The acquired data of 10,488 MS/MS spectra of Strain S1 with maximum toxicity of 60% when searched against the customized toxin database using the software Morpheus led to the identification of 4 toxin protein groups (Supplementary data 2). Those were BinA (PSM: 05, Protein sequence coverage: 15.14% and Morpheus score: 5.11), Cry 57Aa (PSM: 03, Protein sequence coverage: 5.75% and Morpheus score: 5.21), VIP-4 (PSM: 02 Protein sequence coverage: 3.73% and Morpheus score: 4.03), VIP-2 (PSM: 02, Protein sequence coverage: 3.46% and Morpheus score: 4.02) The peptides identified in the proteins of Strain S1 mentioned above are also highlighted in the sequences presented in Supplementary data 2.

Similarly, the acquired data of 9815 MS/MS spectra of Strain S2 when compared with the customized toxin database led to the identification of 14 toxin protein groups as shown in Supplementary data 4. They are Cry 48Ab (PSM: 06; Protein sequence coverage: 9.18% and Morpheus score: 37.19), Cry 70Bb (PSM: 05, Protein sequence coverage: 6.63% and Morpheus score: 25.21), Cry 39Aa (PSM: 04; Protein sequence coverage: 8.49% and Morpheus score: 26.16), Cry 41Ab (PSM: 04, Protein sequence coverage: 6.51% and Morpheus score: 25.09), Cry 30 Da/Ea (PSM: 03, Protein sequence coverage: 4.09% and Morpheus score: 19.13), Cry 49Ab (PSM: 03, Protein sequence coverage: 9.64% and Morpheus score: 17.09), Cry 57Aa (PSM: 03, Protein sequence coverage: 4.27% and Morpheus score: 16.21), Cry 51Aa (PSM: 02, Protein sequence coverage: 15.86% and Morpheus score: 16.16), Cry 35Aa (PSM: 02, Protein sequence coverage: 10.91% and Morpheus score: 14.06), Cry 19Ba/Ca (PSM: 02, Protein sequence coverage: 3.36% and Morpheus score: 13.06), Cry 53Aa (PSM: 02, Protein sequence coverage: 4.33% and Morpheus score: 13.06), Cry 56Aa (PSM: 02, Protein sequence coverage: 6.03% and Morpheus score: 13.04), Cry 33Aa (PSM: 02, Protein sequence coverage: 16.13% and Morpheus score: 11.05) and Cry 64Aa (PSM: 02, Protein sequence coverage: 15.08% and Morpheus score: 10.11). The peptides identified in the proteins of *Bacillus cereus* mentioned above are also highlighted in the sequences presented in Supplementary data 3.

The acquired data of 10,741 MS/MS spectra of Strain S3 when compared with the customized toxin database led to the identification of 20 toxin protein groups as shown in Supplementary data 5. They are Cry 56Aa (PSM: 09; Protein sequence coverage: 23.83%, Morpheus score: 56.23), Cry 25Aa (PSM: 07; Protein sequence coverage: 12.89% and Morpheus score: 45.18), Cry 33Aa (PSM: 07, Protein sequence coverage: 44.19% and Morpheus score: 44.13), Cry 32 Aa/Ba/Da (PSM: 06, Protein sequence coverage: 3.20% and Morpheus score: 23.15), Cry 52Ba (PSM: 05, Protein sequence coverage: 7.68% and Morpheus score: 32.17), Cry 49Ab (PSM: 04, Protein sequence coverage: 12.21% and Morpheus score: 26.16), Cry 30 Da/Ea (PSM: 04; Protein sequence coverage: 5.70% and Morpheus score: 22.11), Cry 32 Ba/Ca (PSM: 04; Protein sequence coverage: 3.64% and Morpheus score: 15.07), Cry 47Aa (PSM: 03; Protein sequence coverage: 4.14% and Morpheus score: 20.16), BinB (PSM: 03; Protein sequence coverage: 17.19%, Morpheus score: 20.06), Cry 51Aa (PSM: 03; Protein sequence coverage: 18.12%, Morpheus score: 18.10), Cry 45Aa (PSM: 03; Protein sequence coverage: 9.82%, Morpheus score: 16.10), Cry 42Aa (PSM: 02; Protein sequence coverage: 5.19%, Morpheus score: 17.05), Cry 39Aa (PSM: 02; Protein sequence coverage: 3.94%, Morpheus score: 16.10), Cry 9Bb (PSM: 02; Protein sequence coverage: 3.78%, Morpheus score: 16.03), Cry 54Aa (PSM: 02; Protein sequence coverage: 4.61%, Morpheus score: 15.08), Cry 38Aa (PSM: 02; Protein sequence coverage: 10.32%, Morpheus score: 14.07), Cry 63Aa (PSM: 02; Protein sequence coverage: 5.31%, Morpheus score: 14.05), Cry 14Aa (PSM: 02; Protein sequence coverage: 2.87%, Morpheus score: 14.05) and Mtx-2 (PSM: 02; Protein sequence coverage: 16.78%, Morpheus score: 13.06). The peptides identified in the proteins of Strain S3 mentioned above are also highlighted in the sequences presented in Supplementary data 4. The concise results of larvicidal protein toxins identified in customized database through LCMS for all the three strains of *Enterococcus* species are shown in Table 3.

**Table 3:** The results of all mosquito-cidal toxins obtained from LCMS after comparing it with the customized database which had all toxin template sequences of Cry (Cry1 to Cry73), Bin (BinA and BinB) VIP (VIP-1, VIP-2, VIP-3, and VIP-4), and Mtx (Mtx1, Mtx2, Mtx3).

S1 (Toxicity-60%)	S2 (Toxicity-35%)	S3 (Toxicity-20%)
Bin-A		
		Bin-B
		Cry 9Bb
		Cry 14Aa
	Cry 19Ba/Ca	
		Cry 24Ba/Ca
		Cry 25Aa
	Cry 30Da/Ea	Cry 30Da/Ea
		Cry 32Aa/Ba/Da
	Cry 33Aa	Cry 33Aa
	Cry 35Aa	
		Cry 38Aa
	Cry 39Aa	Cry 39Aa
	Cry 41Ab	
	Cry 48Ab	Cry 42Aa
		Cry 45Aa
		Cry 47Aa
	Cry 49Ab	Cry 49Ab
	Cry 51Aa	Cry 51Aa
		Cry 52Ba
	Cry 53Aa	
		Cry 54Aa
	Cry 56Aa	Cry 56Aa
Cry 57Aa	Cry 57Aa	
		Cry 63Aa
	Cry 64Aa	
	Cry 70Bb	
		Mtx-2
VIP-2		
VIP-4		

### *In silico* analysis

*In-silic*o analysis of all toxin template sequences of Cry (Cry1 to Cry73), Bin (Bin A and Bin B) VIP (VIP1, VIP2, VIP3, and VIP4), and Mtx (Mtx1, Mtx2, Mtx3) were independently searched against the database of *E. durans* using p-BLAST with default parameters. The toxin sequences with large number of hits and significant E-values (E-values ≤ 10^−10^) were considered. With respect to the above criteria, there were only Cry 7, Cry 8, Cry 22 and Mtx-2 which could be traced back in the protein database of *E. durans*.

After comparing the results of the toxins obtained from the LCMS data with that of the in-silico p-BLAST database of *E. durans*, the presence of only Mtx-2 in the strain S3 were found to be obtained in both the results. This confirms the existence of Mtx-2 in *E. durans*.

### Metabolites and MS data acquisition

The metabolites from all the three strains of *E. durans* were extracted in duplicates and subjected to LCMS for their analysis. The resulting data of the plausible metabolites obtained were briefly analyzed. The metabolites having a score of more than 99.5% were selected and reported. As per this selection criteria, LCMS data of the metabolites resulted in the acquisition of total 63 and 40 metabolites in Set A and Set B, respectively for the strain S1, out of which 13 metabolites were found to be commonly present in both the sets. Similarly, the strain S2 resulted in acquisition of 46 and 49 metabolites in Set A and Set B respectively, out of which, 16 were common metabolites. Lastly, the strain S3 procured 53 and 39 metabolites in Set A and Set B respectively with 18 of them as common. The complete details and list of the attained metabolites are shown in the Supplementary data sheet 3. The 13, 16 and 18 common metabolites obtained from each of these three strains S1, S2 and S3 respectively, were compared and studied for their role in various biochemical pathways using the online tool KEGG (https://www.genome.jp/kegg/pathway.html). The metabolites which are found common in all three of the *Enterococcus sp*. are terephthalic acid, C16 Sphinganine and Phenyl methyl ketone. The ones present in atleast two of the strains were N-(2-hydroxyethyl) icosanamide; 22-methyl-tricosanoic acid; Trolamine; 2,2,9,9-tetramethyl-undecan-1,10-diol; 3beta,6alpha,7beta-Trihydroxy-5beta-cholan-24-oic acid Isopentadecyclic acid, octacosanal and 4,8-dimethyl-dodecanoic acid. These common metabolites are highlighted in different colors in Supplementary data sheet 3.

### ^1^H NMR of extracted metabolites

**Figure 3:**
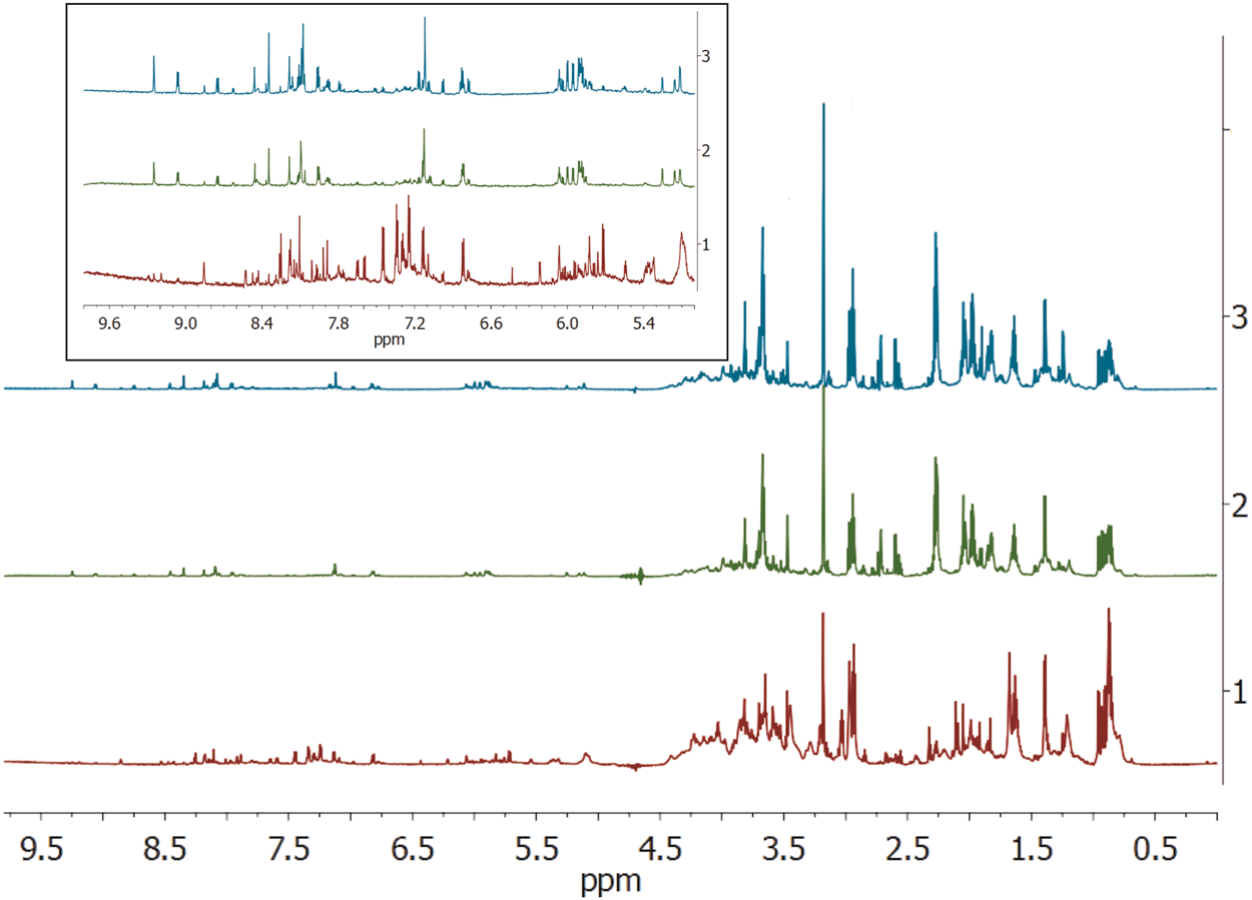
The NMR spectra of the extracted metabolites obtained from Bruker 800 MHz AVANCE-NEO (Topspin 4.01).

The metabolites of all the three strains of *E. durans* were extracted using acetonitrile and subjected to 1D ^1^H NMR to compare the metabolomic profiles of the strains. The NMR spectra of these three strains ranging from 0.5 ppm to 9.5 ppm are shown in Fig. 3, whereas the inset shows the expanded NMR spectra of the same ranging from 5.4 ppm to 9.5 ppm. The nature of the NMR spectra of metabolites from all the three strains, are more or less similar with very slight variation in the maximum toxic strain S1 at the aromatic regions between 6.5 ppm to 8.4 ppm^11^. Also, there are differential peak patterns between 5.5 & 6.5 ppm. Identification of these metabolites using NMR spiking is beyond the scope of this manuscript.

### Cytotoxicity using MTT Assay

**Figure 4:**
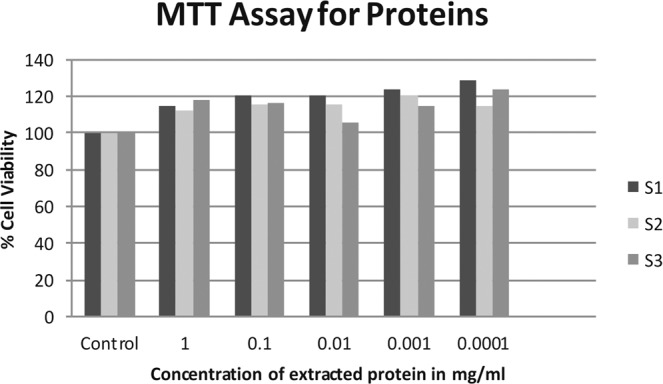
The MTT Assay of the extracted proteins showing % cell viability of L-929 cell lines.

**Figure 5:**
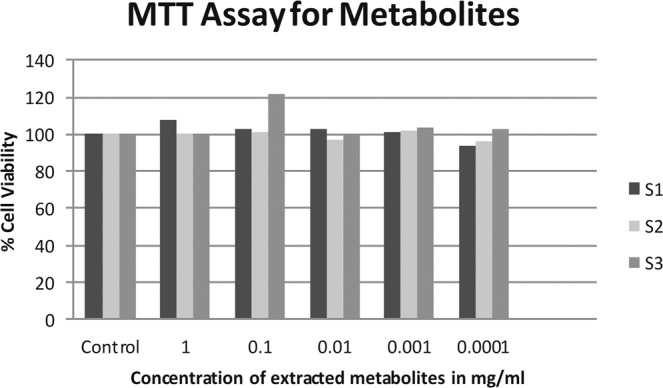
The MTT Assay of the extracted metabolites showing % cell viability of L-929 cell lines.

The extracted proteins and metabolites were exposed to the L-929 (mouse lung fibroblast) cell lines for testing their cytotoxicity against the animals. The absorbance obtained after the MTT assay are shown in Supplementary data Sheet 4. These absorbance data of the test was co related with the viability of the control cells. The percent cell viability of the extracted proteins and metabolites at different concentration ranging from 1 to 0.0001 mg/ ml are shown in Figs. 4 and 5 respectively. It was observed that the isolated proteins showed a cell viability of more than 100% and the metabolites showed cell viability of not less than 90% at different concentrations. Thus, the extracted proteins and metabolites from our three strains of *E. durans* are safe and not responsible for showing any kind of toxicity on the eukaryotic cell lines.

(mg/ml: milligram per milliliter)

## Discussion

The three bacterial strains used in this study, were isolated from the mid-gut of dead *C. quinquefasciatus* mosquito larvae collected from the wild. They were tested for their toxicity against the third instar laboratory cultured *Culex* mosquito larvae, which recognized them to possess differential toxicities ranging from a minimum of 20% for strain S3, intermediate 35% for strain S2 and a maximal of 60% for strain S1. The larvicidal activity in terms of LC_50_ values against *C. quinquefasciatus* was and 59.63 CFU/ml for Strain S3, 48.43 CFU/ml for Strain S2 and 35.67 CFU/ml for Strain S1 as shown in Table 1 where CFU/ ml – colony forming units per milliliter. These LC_50_ values on conversion corresponds to 59.63 × 10^−3^ ng/ml for S3; 48.43 × 10^−3^ ng/ml for S2 and 35.67 × 10^−3^ ng/ml for S1, if bacterial mass of 1 CFU is considered to be 1 pico- gram^6,7^. These LC_50_ values reflect that our *Enterococcus* strains are almost 100 times more potent as compared to the toxicity shown by well-known *Bt* which ranges from 0.5 to 1.47 ng/ml^12^. These findings allowed us to report *Enterococcus sp*. for the first time for having substantial mosquito larvicidal toxicity apart from the very well-known *Bacillus sp*.

Upon identification by 16S rRNA sequencing technique, all three of these bacteria were found to be strains of *E. durans* which were further imaged by Scanning Electron Microscope for their (cocci) spherical morphology, which additionally supports our sequencing results.

The distinctive characteristic of exhibiting differential toxicity by the three same strains of *E. durans* was elucidated using proteomic and metabolomic approach using mass spectrometry. In our data acquisition for the proteomic components, the LCMS acquired 10,488 MS/MS spectral peaks for the maximum toxic (60%) strain S1, 9815 MS/MS spectra for medial toxic (35%) Strain S2 and 10,741 MS/MS spectra for Strain S3 which had the least toxicity of 20%. Through our LCMS analysis, we could successfully identify 4 peptides belonging to 2 isoforms of VIP and one each of Binary and Cry toxins for strain S1; 14 peptides all belonging to different isoforms of Cry toxins for Strain S2 and 20 peptides belonging to 18 isoforms of Cry and one each of Bin and Mtx toxins for strain S3. The Table 3 shows all the toxins obtained in our analysis for the three respective strains.

As is quite evident from the Table 3, the toxicity of strain S1 and S3 is not likely due to the Binary toxin system of BinA and BinB as it is well established that the binary toxins BinA-BinB of *Lysinibacillus sphaericus* (*Bs*) showed maximum toxicity against *Culex* larvae when functions as a hetero-dimer in stoichiometric mixture of 1:1^13^. This shows that the two components are active only when they are co-expressed in equal proportions and hence both should have been detected in our spectra. In strain S3, we found only Bin B which happens to be the inactive component of the complex. However, in our maximum toxic strain S1, we obtained only Bin A whose activity seems to be compromised in the absence of its interactive partner, Bin B^13^. Our data analysis revealed no presence for Binary toxins in Strain S2.

For our maximum toxic strain S1 we also found the presence of VIP-2 and VIP-4 which were not found in any other strains. But these VIP toxins are also not responsible for the toxicity of strain S1 because, like Binary toxins, VIP-1 and VIP-2 exhibits high insecticidal activity against some coleopteran pests^14^. But their homology to other bacterial binary toxins suggests that VIP-1 and VIP-2 form typical A + B type binary toxins, where VIP-2 has the cytotoxic A-domain and VIP-1, the receptor-binding domain, is responsible for the translocation of the cytotoxic VIP-2 into the host cell^15^. The presence of only VIP-2 in the strain S1 makes it the irrelevant candidate for toxicity. Nothing can be commented about the activity of VIP-4 since it is a recently reported to the toxin family with no target insects^15^. Finally, the non- availability of any literature for Cry 57Aa makes it difficult to comment about its role in the toxicity of the strain S1.

Similar to the function of Binary and VIP toxins, there were two more groups of cry toxins which were found to exhibit a two-component dependency. These were Cry 48Aa1 - Cry 49Aa1 and Cry 35 - Cry 36^16^. The strain S3 was found to have only one of these two component; namely Cry 49 alone. Hence, this toxin can also may not be the primary reason for its toxicity. Since, the strain S2 shows presence of both Cry48 & Cry 49, it can be a fair reason for its toxicity whereas presence of only Cry35 with absence of its potential partner Cry 36, reduces its chances to be active.

For our second most toxic strain S2 with 35% toxicity, we found only cry toxins in our LCMS data. The Cry 19 are known to be toxic against animals of dipteran group^14^. A novel crystal protein, Cry 30Fa1 and Cry 54Aa1 was cloned and characterized from *Bt* strain BtMC28 and was known to have toxicity against *Laphygma exigua* (Lepidoptera), *Helicoverpa armigera* (Lepidoptera), and *Aedes aegypti* (Diptera)^17^. The Cry 33, Cry 51 and Cry 64 show features which are similar to Mtx-2 toxins^18^. These are called Epsilon toxins (ETX_MTX2) which are produced by strains of *Clostridium perfringens*. It belongs to the heptameric β-pore-forming toxins including aerolysin and *Clostridium septicum* alpha toxin, which are characterized by the formation of a pore through the plasma membrane of the host eukaryotic cells^19^. Some para-sporal crystals of *Bt* are termed as para-sporins such as Cry31A, Cry41A, Cry45A, Cry46A, Cry63A and Cry64A alternatively named parasporin-1 (PS1), parasporin-3 (PS3), parasporin-4 (PS4), parasporin-2 (PS2), parasporin-6 (PS6), and parasporin-5 (PS5), respectively. They are known to exhibit strong and specific cytocidal activity against human cancer cells of various origins^20,21^.

The least toxic strain S3 with 20% toxicity, showed the presence of 18 isoforms of Cry toxins and Mtx-2. As discussed earlier, Cry15, Cry23, Cry33, Cry38, Cry45 (parasporin 4), Cry51, Cry60 and Cry64 all show features of the ETX_MTX2 family that includes the Mtx2 protein from *Bs* and the Clostridium epsilon toxin^18,19^. A very recent report says that, when applied together, the three-domain Cry toxin, Cry9Aa and the VIP, VIP3Aa, exhibited high insecticidal activity against an important insect pest, the Asiatic rice borer (*Chilo suppressalis*)^22^. Similarly, Cry 9 is known to have toxicity against some lepidopteran^14,23^. Some protein are known to have no toxicity and can enhance the activity of other proteins. Cry 14 has similar kind of activity of enhancing the toxicity of Cry 15 and Cry 33^24^. Since Cry 14 and Cry 33 both are present in Strain S3, they can also be the potential reason for their host toxicity. The toxicity of Cry 30Fa1 and Cry 54Aa1 from *Bt* strain BtMC28 toxic to *Aedes aegypti* mosquito are mentioned earlier^17^ were both found to be present in strain S3. The Cry 24, Cry 32 and Cry 47 are known to be toxic against animals belonging to the dipteran family^14^.

The commonly expressed toxins of Cry30, Cry 33, Cry 39 and Cry 56 in strains S2 and S3; and Cry 57 obtained in strains S1 and S2 can be ruled out for their potential roles in toxicities as we observed differential levels of mosquito-cidal activities for same strains of *Enterococcus* (S1 and S2). For the other candidates of the Cry family, i.e., Cry 25, Cry 39, Cry 42, Cry 52, Cry 53, Cry 56, Cry 57, and Cry 63, we could not find any concrete literature which could link these identified proteins to larvicidal activity. This makes them as novel targets that needs to be explored further by the scientific fraternity.

It is quite interesting to note that the LCMS proteome data of our maximum toxic *Enterococcus* strain S1 resulted in the detection of very few mosquito-cidal toxins, although the number of MS/MS spectral peaks identified in all the three samples were almost same. This calls upon a need for looking at alternate toxins in the know category or a novel category all together, i.e., apart from what is known in the literature and has been used to prepare our customized database. These novel toxin(s) are either capable of functioning at such low concentration that they are not explicitly detected on mass-spectrometric analysis; or these levels of toxicity are due to the presence of differential metabolites as is explained in the subsequent section.

We undertook a second parallel approach of differential metabolomics where the metabolites extracted from all the three strains were subjected to LCMS and the results obtained above 99.5% confidence limit were considered for analysis. It is quite evident from Supplementary data Sheet 3 that the metabolites common to all the three or at least two of the strains doesn’t seem to have any role in larvicidal activity. In addition, various forms of higher fatty acids and alcohols also seem to be unlikely candidates, altering toxicity, as various forms of these category of compounds are commonly found in all the three strains. However, our 800 MHz proton NMR spectra of the metabolites from the three strains are differential in nature and particularly the strain with highest toxicity (S1) has prominent differences in the aromatic region (6.5 and 8.5 ppm) of NMR. It should be noted that both our LCMS and NMR results are not sufficient to pinpoint the exact nature of the metabolite responsible for the differential mosquito larvicidal nature of the three *Enterococcus* strains. Identifying the right metabolite using NMR will involve rigorous spiking experiments, which is beyond the scope of this manuscript and will be reported elsewhere.

After elucidating the metabolites by LCMS and NMR, it is quite evident that in addition to the proteome, even the metabolome of the *E. durans* needs to be considered while commenting on the differential toxicity. However, the possible role of the metabolites in the mosquito larvae-cidal activity requires further investigation as very little data is available in the literature on these lines.

Also, these proteins and metabolites extracted from our strains were tested for their cytotoxicity against the L-929 cell lines. These experiments confirm that for either of the components, there is no adverse effect on the viability of the eukaryotic cell line. These results in addition to our larvicidal toxicity data helps us to propose our *E. durans* strains as a novel eco-friendly and bio-friendly alternative to be used as biopesticide against *Culex* mosquito larvae.

The genus *Enterococcus* is a member of Lactic acid bacteria (LAB) which constitutes a part of human associated microbiota in mouth, skin and intestine. They have many interesting properties such as multi-bacteriocin production, probiotics and as a viable alternative to antibiotics^25^. Generally, the bacteriocins produced by the LAB family members are considered safe^26–28^. On the basis of their thermal stability these bacteriocins are classified into three main classes; namely, class I (lantibiotics), class II containing small thermostable antimicrobial peptides, and class III large heat labile bacteriocins. Till date only four bacteriocins, all belonging to the class II have been reported for *E. durans* and they are as follows: durancin TW-49M homologous to enterocin B^26^, durancin GL produced by *E. durans* 41D^27^, durancin L28-1A^28^, and peptides A5-11A and A5-11B with a high degree of similarity to enterocins L50A and L50B^29^ respectively. More recently, a bacteriocin-like substance produced by *E. durans* E204 was reported as an inhibitor for the growth of *L. monocytogenes* in cheese^30^. Likewise, application of enterocins AS-48 and CRL35 inhibited pathogenic microorganisms such as *S. aureus, L. monocytogenes* and thereby prevents the spoilage of different dairy products^31^. All of these toxin history of *E. durans* justifies our claim to consider *E. durans* as a safe alternative to control *Culex* mosquito population.

In spite of their ability to exhibit pathogenic activity, these symbiotic *Enterococci* generally are generally non-virulent or show minimal virulence. This is quite evident as they are established natural colonizers of the gastrointestinal tract of humans and animals. Also, these bacteria have been used safely for centuries and decades as probiotics in the diet of humans and farm animals^32^. They are vital for the dairy industry and are commonly used as non-starter LABs in cheese. *E. durans* and *E. faecalis* play an essential role in the maturation of different cheese varieties. This could very likely be because of their proteolytic or lipolytic activity and also because of their ability to ferment citrate to produce diacetyl and other volatile compounds that is responsible for a characteristic flavor and taste^25,33^. Moreover, some enterococci have been known to possess anti-carcinogenic, hypo-cholesterolemic, as well as immune regulation effects. For instance, *E. durans* M4-5 has been known to produce butyrate, a short chain fatty acids (SCFAs), that induces significant anti-inflammatory effects and also is one of the important components that contribute to the integrity of the intestinal epithelium^34^. *E. durans* KLDS 6.0930 has been widely used as a probiotic candidate which also has a capability to lower human serum cholesterol levels^35^. Finally, *E. durans* LAB18s is well known as a dietary source for selenium supplementation^36^.

## Conclusions

This study made an attempt in identifying *E. durans* as a potential mosquito-cidal strain which, to the best of our knowledge, has not been reported earlier for having mosquito larvicidal activity. Also, we are the first to report the proteomic and metabolomics profiles of *E. durans*. The proteomic and metabolomic investigation described in this manuscript provides for the first time a comprehensive evaluation to reveal the plausible reason for the differential toxicities. At the proteomics level, we tried to identify various proteins which we could correlate to larvicidal toxin activity in *E. durans* and our data helped us to characterize plausible mosquito larvicidal proteins. The para-sporin nature of Cry31A, Cry41A, Cry45A, Cry63A and Cry64A; the Epsilon related toxins Mtx-2, Cry 33, Cry 51 and Cry 64; the correlation potency of Cry 35-Cry36 and Cry 48-Cry49 and finally the toxicity of Cry 24, Cry 30, Cry 32, Cry 39, Cry 49 and Cry 50 against the dipteran class of animals (to which even mosquitoes belong) can be the plausible toxins responsible for the toxicity in our obtained strains of *E. durans*.

The NMR spectra of the metabolites showed more number of peaks in the aromatic region for the strain exhibiting highest mosquito larvicidal activity, which helped us to elucidate that the differential toxicity within the same species of *Enterococcus* could be a characteristic function of their differential metabolome. The NMR data along with the LCMS data of metabolites which detected unsaturated fatty acids and complex alcohols, if not toxins themselves, but could be the potential regulators for triggering the release of mosquito-cidal toxins. Hence, we cannot rule out the possibility of their role in toxicity which needs further investigation and analysis. This one of a kind study also showed that the differential proteomics must be complemented with their differential metabolomics to interpret the differential toxicities. This manifest into that the release of toxins might be related with the regulation of the metabolites as well, and not subjected to the proteome alone.

Since, *Enterococcus sp*. have attracted great research interest as a natural antimicrobial agent in the food industry, and as a potential drug candidate for replacing antibiotics in order to treat multiple drug-resistance pathogens. Our present study reports *E. durans*, for the first time to our knowledge, to show highly potent mosquito larvicidal activity. The mosquito-cidal toxicity of these strains against *C. quinquefasciatus* are much more potent than the already used bacterial strains to which mosquitoes are getting quite resistant. Nonetheless, the proteome and metabolome of our isolated strains were also found to be having no cyto-cidal effect on eukaryotic animal cells in our MTT assay which makes *E. durans* safe to be used in the wild environment. Being non-toxic to environment, mosquito-cidal and probiotic history, these can be used as an eco-friendly and bio-friendly alternative, complementary or entirely substituting the already well-studied *Bt* and *Bs* strains for mosquito control.

## Methods

All the reagents used in the study were from Sigma Aldrich or else mentioned.

### Collection of mosquito larvae & screening of midgut microflora

Dead mosquito larvae of *C. quinquefasciatus* in their fourth instar stages were collected from various locations of Western Mumbai, India. The position co-ordinates of these locations are provided in the Supplementary data 1. After collecting these dead mosquito larvae, they were surface sterilized using ethanol and their mid-gut was dissected under the compound microscope. The complete gut material was suspended in Luria Bertini media which was later used for plating on gram-positive selection media like Columbia CN (Colistin, Nalidixic acid) Agar^37^, Phenyl-ethanol Agar^37^ and a US Patented media^38^.

### Toxicity assay for mosquito larvicidal activity

The gram-positive bacteria which were screened on the selective media were individually tested on F1 generation of laboratory cultured, third instar stage of *C. quinquefasciatus* mosquito larvae for their toxicity. Prior to the toxicity assay, the test larvae were starved overnight and then ten of them were suspended in the 24-hour old culture of the screened gram-positive bacteria at dilutions of 1:5; 1:10; 1:100 and 1:1000. The total volume in every test were kept constant at 30 ml. Two sets of control, one with mosquito larvae feed and another without the feed were used. Control with larvae feed was kept to avoid standard error in mortality due to starvation. Only those experiments were considered wherever both these controls should no deaths. The percent mortality out of the ten larvae was observed in every test and control after 48 hours^39^. All the tests and controls were performed in duplicates and the readings are average of three independent experiments. The average of the mortality data was subjected to probit analysis for calculating LC_50_ at 95% confidence limit (LCL) using the SPSS 16.0 version (Statistical software package) to find the regression equation values. Results with p ≤ 0.05 were considered to be statistically significant^40^.

### Identification of toxic bacteria by DNA extraction and 16S rRNA sequencing

For identification, the bacteria were cultured overnight in Luria Bertini media and then subjected to their DNA isolation and purification the following day using HiPurABacterial Genomic DNA Purification Spin Kit (HIMEDIA). The three bacterial strains were identified by 16S rRNA sequencing using five primers 27F, 1492R^41,42^, 704F, 907R and 519R^43^ (Lutzoni Lab- Lichenology and Phylogenetics) at the National Centre for Microbial Resources, (NCMR) - National Centre for Cell Science (NCCS- Pune, India). These sequencing results were analyzed and identified using the NCBI 16S rRNA bacterial database (https://blast.ncbi.nlm.nih.gov/Blast).

### SEM imaging of bacteria

The study of surface morphology of the identified bacteria was done by imaging using Scanning Electron Microscopy (SEM). The microscope used was ZEISS Ultra-field Emission SEM and ZEISS Environmental SEM at TIFR, Mumbai, India. The initial sample preparation of the bacteria used for imaging included four steps of fixation (3% glutaraldehyde); phosphate buffer washings followed by ethanol dehydration and lastly critical point drying prior to mounting the specimen at room temperature^44^ (Standard preparation of biological material for SEM analysis. 500).

### Protein extraction

The bacterial colony was inoculated in 200 ml of Luria Bertini media and incubated at 37 °C overnight. The following day, cells were pelleted by centrifuging at 13000 rpm for 15 minutes at 4 °C. The pelleted cells were then sonicated at amplitude 30%, pulse 10 seconds, gap 10 seconds, for a total time of 2 minutes: 30 seconds or 15 Cycles to obtain the cell lysate. The protein extraction was done using this cell lysate by TRIzol method with minor modifications^45^. Briefly, 300 µL of cell lysate was mixed vigorously with 1 ml of TRIzol reagent and 200 µL of chloroform and then allowed to stand still for 5 minutes at room temperature to obtain a three-phase layer. Centrifugation was carried out at 14,000 g for 15 minutes at 4 °C to separate the three layers distinctly. Upper clear phase was removed carefully and discarded. Around 300 µL of ethanol was added to the remaining solution containing DNA and protein mixture and incubated at room temperature for 3 minutes. The DNA was pelleted by centrifugation at 2000g for 15 minutes at 4 °C. The supernatant comprising of the protein was further purified by addition of 4-fold volume of ice-cold acetone (~1 ml) and incubated overnight at −20 °C. On the next day, protein pellet was thoroughly washed first with 0.3 M Guanidine-Chloride in 95% ethanol thrice and then twice with ice-cold acetone. After air-drying, the protein pellet was dissolved in rehydration buffer (7 M urea, 2 M thiourea, 2% CHAPS, Distilled water). Protein quantification was performed using Bradford reagent (BIO RAD) with BSA as a standard.

### In solution trypsin digestion

The protein extracted by the above mentioned TRIzol method was digested using trypsin (PROMEGA). Briefly 15 µL of Digestion Buffer (50 mM Ammonium bicarbonate) and 1.5 µL of Reducing Buffer (100 mM Dithiothreitol) were added to 10uL of the extracted protein solution. The final volume was adjusted to 27 µL with ultrapure water. This solution was incubated at 56 °C for 45 minutes and allowed to cool at room temperature. Then 3 µL of freshly prepared alkylation buffer (100 mM Iodoacetamide) was added and kept for incubation in the dark at room temperature for 20 minutes. Finally, 10 µL of Trypsin (20 µg/ml) solution was added and kept for incubation at 37 °C for 15 hours (overnight)^46^.

### De-salting of trypsin digested peptides

Desalting of trypsin digested peptides from the above step was optimized and performed using octadecyl carbon chain (C18) pipette tips (ZIPTIP PIPETTE TIPS, MILLIPORE). Briefly, the desalting C18 pipette tip was pre-equilibrated using a solution of 50% Acetonitrile (SRL) in LC-MS grade water and then equilibrated twice in 0.1% formic acid in LC-MS water. After carefully and gently pipetting the entire sample up and down for at least 10 times, the membrane was washed with 0.1% formic acid in LC-MS water. The peptides were eluted in 70% acetonitrile in LC-MS grade water and vacuum dried and stored for LCMS analysis^47^.

### Extraction of metabolites

The overnight grown bacterial culture in 200 ml Luria Bertini media was used for metabolite extraction. The cells were pelleted the following day by centrifugation and the pellet was given sterilize water washings to remove traces of remaining media. Then the bacterial cell pellets were sonicated at amplitude 30%, pulse 10 seconds, gap 10 seconds, for a total time of 2 minutes: 30 seconds or 15 Cycles in 300 µL of 1x Phosphate Buffer Saline (PBS). Post-sonication, the samples were centrifuged at 12000 rpm for 20 minutes and the supernatant was collected in fresh tubes. The metabolites in the supernatant were precipitated by adding acetonitrile in twice the volume and then incubating at −80 °C overnight. The clear supernatant was centrifuged and transferred into new tubes for air drying^48^. These dried samples were further given for LCMS.

### LC-MS/MS analysis

The mass spectrometric analysis was carried out by nano-LCMS using 6540 UHD Accurate Mass QTOF (CAMS, Venture Center, National Chemical Laboratory- Pune, India). The peptides were analyzed using reversed phase nano-scale liquid chromatography coupled to tandem mass spectrometry. The system consisted of a column; initially the sample was subjected to Agilent Binary LC1290 using column Polaris, high performance chip, 360 nano-liter enrichment chip (150 mm × 75 µm) and a separation column G4240–62030 with a thermostat temperature of 4 °C. The mobile phase was Water (0.1% Formic Acid) and Acetonitrile: Water (90:10 v/v). The chromatography was carried out in a gradient manner (3% to 90% Acetonitrile: Water). The mass spectrometry analysis was carried out on Agilent 6540 UHD QTOF MS (Gas temp: 250, Gas flow (l/minute): 8 minutes,) MS analysis was carried out in a data dependent manner with survey scan resolution. Mass range: 250–1700(m/z), MS/MS: 50–1700(m/z), Data type: Centroid and software: Mass hunter workstation software. Obtained data was searched against the UniProt protein databases of *E. durans* species using default parameters.

### Database preparation and search for protein identification

The data obtained from high resolution MS/MS spectra was searched against the customized mosquito larvicidal toxin database (Cry1 to Cry73, Bin-A/B, VIP-1/2/3/4 and Mtx-1/2/3) incorporated with the Morpheus software suite^10^. The default search parameters used for the analysis are as follows: (a) trypsin as the proteolytic enzyme with up to one missed cleavage; (b) peptide mass error tolerance of 20ppm; (c) fragment mass error tolerance of 0.1 Da; (d) carb-amido-methylation of cysteine as fixed modification (e) oxidation of methionine as a variable modification. A false discovery rate of 1% was applied while identifying the peptide-spectrum matches. Two criteria used to report a positive find, (1) reported protein/toxin should have least two numbers of peptides in amino acid sequence; and (2) the length of each of the identified peptide sequence using LC-MS has a minimum of 10 amino acids.

### *In-silico* analysis

In order to find the existence of the known mosquito larvicidal toxins in our identified *Enterococcus species*, the National Center for Biotechnology Information (NCBI) database was used for in-silico analysis. The sequences of all the known mosquito-cidal toxins like Crystal (Cry1 to Cry73) proteins with the Vegetative insecticidal proteins (VIP-1, VIP-2, VIP-3, VIP-4) from *Bt* and the Binary (Bin-A and Bin-B) toxins with the Mosquito-cidal (Mtx-1; Mtx-2; Mtx-3) toxins from *Bs* were used as template sequences to carry out p-BLAST search within the databases of our identified *E. durans* with default search parameters. (https://blast.ncbi.nlm.nih.gov).

### ^1^H NMR of extracted metabolites

The extracted metabolites of all the three *E. durans* species were subjected to ^1^H NMR to understand the difference in the metabolome. All the one-dimensional NMR experiments were performed on Bruker AVANCE-NEO (Topspin 4.01) 800 MHz spectrometer equipped with cryo-cooled probe^49^ at National Facility for High-Field NMR in Tata Institute of Fundamental Research (TIFR), Mumbai, India.

### Cytotoxicity using MTT assay

A panel of NCTC clone 929 [L cell, L-929, derivative of strain L] cell lines obtained from National Center for Cell Sciences (NCCS-Pune) were seeded into 96-well flat bottom microtiter plate (HIMEDIA) at a density of 5 × 10^3^ cells per well and allowed to adhere for 24 hours at 37 °C in a CO_2_ incubator. After 24 hours of incubation, culture medium was replaced with a fresh Dulbecco’s Modified Eagle’s medium-DMEM and treated with various concentrations of the extracted protein and metabolites for 24 hours at 37 °C in a CO_2_ incubator. Measurement of cell proliferation was monitored by the NAD(P)H-dependent cellular oxido-reductase enzyme in viable cells converting the yellow tetrazolium MTT [3-(4, 5-dimethylthiazolyl-2)-2, 5-diphenyltetrazolium bromide] into insoluble (E,Z)-5-(4,5-dimethylthiazol-2-yl)-1,3-diphenylformazan [formazan]. This formazan can be dissolved with dimethyl sulfoxide to give purple color with characteristic absorption at 595 nm. The purple coloration is directly proportional to cell viability. The cells were exposed to MTT for 4 hours and absorbance was measured using a microplate reader (Tecan Infinite M200) at 595 nm^50^.

## Supplementary information

Supplementary data.

Supplementary datasheet 1.

Supplementary datasheet 2.

Supplementary datasheet 3.

Supplementary datasheet 4.
