# Supplementary material for: Enterococcus durans with mosquito larvicidal toxicity against Culex quinquefasciatus, elucidated using a Proteomic and Metabolomic approach
Source: Sci Rep. 2020 Mar 16;10:4774. doi: 10.1038/s41598-020-61245-2 (PMC7075886; doi:10.1038/s41598-020-61245-2)

***Enterococcus durans* with mosquito larvicidal toxicity against** ***Culex quinquefasciatus,* elucidated using a Proteomic and Metabolomic approach**

Domnic Colvin, Vishnu Dhuri_,_ Hriday Verma, Rama Lokhande, and Avinash Kale

**Supplementary data**

**Supplementary data 1:**

| **Site** | **Latitude** | **Longitude** | **Strains** | **Identified Species** |
| --- | --- | --- | --- | --- |
| 1 | 19°N 18ʹ 44.293ʺ | 72°E 50ʹ 59.868ʺ | S1 | *Enterococcus durans* |
| 2 | 19°N 27ʹ 20.8ʺ | 72°E 48ʹ 48.1ʺ | S2  **and**  S3 | *Enterococcus durans* |

**Supplementary data 1:** The location co-ordinates for the collection sites from where the dead Culex mosquito larvae were collected.

| **Sr. No.** | **Protein Name** | **Number of Peptide-Spectrum Matches** | **Summed Unique Peptide Precursor Intensity** | **Protein Sequence Coverage (%)** | **Summed Morpheus Score** |
| --- | --- | --- | --- | --- | --- |
| 1 | Bin A | 5 | 34816.92 | 15.14 | 5.11 |
| 2 | Cry 57Aa | 3 | 5483.93 | 5.75 | 5.21 |
| 3 | VIP-4 | 2 | 6340.03 | 3.73 | 4.03 |
| 4 | VIP-2 | 2 | 6222.44 | 3.46 | 4.02 |

**Supplementary Data 2**: Proteome data of the toxin proteins identified in customized database from 10,488 MS/MS spectra of S1

**RESULTS**

5,982 total (2,991 target + 0 decoy + 2,991 on-the-fly decoy) proteins

598,541 total (299,179 target + 299,362 decoy) non-unique peptides

10,488 MS/MS spectra

1,972 target (18 decoy) PSMs at 0.912% PSM FDR (9.031 Morpheus score threshold)

1,105 unique target (11 decoy) peptides at 0.995% unique peptide FDR (9.036 Morpheus score threshold)

246 target (2 decoy) protein groups at 0.813% protein group FDR (9.096 summed Morpheus score threshold)

0.77 minutes to analyze

The unique peptides identified in the proteins of S1 corresponding to larvicidal toxins are highlighted in the sequences

1. >BinA gi|112297518|gb|ABI15165.1| **binary toxin A [Lysinibacillus sphaericus]**

MRNLDFIDSFIPTEGKYIRVMDFYNSEYPFCIHAPSAPNGDIMTEICNRENNQYFIFFPTDDGRVIIANR

HNGSVFTGEATSVVSDIYTGSPLQFFREVKRTMATYYLAIQNPESATDVRALETHSHELPSRLYYTNNIE

NNSNILISNKEQIYLTLPSLPENEQYPKTPVLSGIDDIGPNQSEKSIIGSTLIPCIMVSDFISLGERMKT

TPYYYVKHTQYWQSMWSALFPPGSKETKTEKSGITDTSQISMTDGINVSIGADFGLRFGNKTFGIKGGFT

YDTKTQITNTSQLLIETTYTREYTNTENFPVRYTGYVLASEFTLHRSDGTQVNTIPWVALNDNYTTIARY

PHFASEPLLGNTKIITDDQN

1. >cry57Aa gi|225348555|gb|ACN87261.1| Cry delta-endotoxin [Bacillus thuringiensis serovar kim]

MGTWWPTDSASDTWGEMIGFAQELVGTALSEDLKIRTNQQIDSIRIALQAYYSSLEDWLNANKPLSGPLL

NQVTEEFGNALRKSRDSIAYFKSDDSNVYTIILLPAYAQVANFHLALIHEGLKYATEWNLPRLQTFGYEE

DLKHYTISYVNHCEYWYQKGLDILYPRNVIGMTQWMKRNFYRLNMTINVLDIISLFSLYDSKKYPNFFED

IYNAQKELISKFQLTRIVTTEPTLHQHKYLNDSSKKICQNESSCDPIDLDEYLTLPLMFQNWLRNINFQY

LAPVISVGDEALYPFFVATQNINEYMNAEGNMIIGRQQGLWNFQFIIVHLFHLVYKKMTIFMVKWLSAYP

LIDEDILNGVRTPYILQKIEFYNLNKSINSKQIRPISAGTTKSPLIDIYYGLPDVNGYNLDEPQFNFNAA

SHYFNSIQTCYYKENTSKNHYDIYQSYVFHWEHASVKRKDEVVSDR.ITIFPAIKSNPILSRGIQIISHQGHTGGNVIYFTPQSELHFKINFVSNRQKYKIRLRYVAFNPVVIQYHGSNSYASLSSITLPRTSSNQNVRDLRYEEFGYSDFEINMSSAGGLEDIKIISNNEFILDRIEFIPDTLFNYLSN

1. >gb|WP_000769760.1|WP_000769760.1 vip4

MKLKSTFKCLTITAVLSQITVYPTTSYAENIDRTINTDKSKEEQNSQGLLGYHFKDNQFEKLSYIEVGIKNKEEEKKQRMKRSIEDEKNLSIQSVRWLGRLVVPETGEYTLSTSFDQHVILQINGETVLNKGKTVKSVSLEKDKAYEVKIEYQNTENIETDLQLFWSINGQDKKLIPHQNIVSPDFSKKENLPEDKLNTALIPNSNLFNGKASSTNMEDTDQDGIPNEWEEKGYTFKNQQIVKWDDSYLSQGYKKYLSNPYKARTIADPYTDFEKVSGHMPAATKEDARDPLVAAYPAVGVGMENLLFSKNENVTEGSSGTMSKSVTDTNTNTNNVDLSAKLGWNDKGFGFEFTPKYSHTWTNSTAVQNSESESWSSQVGINSAESAYLNANVRYYNAGTAPIYDLKPTTNFVLQNSGKSLATITAGPNQIGNSLGPGDTYPKVGQAPISLDKANDAGTVKIPINKDYLDALQSNSEALDLETTQNKGQYGVLDATGQLITDSSKQWDPVRTNIDSVSGSLTLNLGSSKESLERRVAAKNDDDPEDKTPEITIGEAIKKAFNAKEKDGRLYYVNSNGENVFLDESSVNLIGDENTKKDIEQQLEHMEDKKVYNAKWKRGMKITIHVPTSYYDFEKSGDSQWYNTYQDNGGYTGEKTGRINPGSNGYAIKDFTLKPYTSYTARAYVKASSSETDAVFYVDSDINSIGKGIKQNIKATGDKWKLVEMSFNTGSNPELFKKVGFKNQGNVQLQFDDVSVTEWKTEENLEKTHSMENWDVDPSKQYVKGGTFSHVPNSKIRYQWKINDNWEKIIPAPPVDNYGKRVMEKNFNFNDHVELYAVDEHNDYLKVKVAEHNKGDAITEDVLKSSHQFSTWIKSKAPGGGSYTDGWYFERIPDGVLHCVTKYKVSINGGKPVTRDRYNPDKNGRMEVNLLEYNGGRGVKEGSRIEAWAILSNGKEAKVLDKKTS

1. >gb|1QS1_A| 1QS1_A vip 2

MKRMEGKLFMMSKKLQVVTKTVLLSTVFSISLLNNEVIKAEQLNINSQSKYTNLQNLKITDKVEDFKEDKEKAKEWGKEKEKEWKLTATEKGKMNNFLDNKNDIKTNYKEITFSMAGSFEDEIKDLKEIDKMFDKTNLSNSIITYKNVEPTTIGFNKSLTEGNTINSDAMAQFKEQFLDRDIKFDSYLDTHLTAQQVSSKERVILKVTVPSGKGSTTPTKAGVILNNSEYKMLIDNGYMVHVDKVSKVVKKGVECLQIEGTLKKSLDFKNDINAEAHSWGMKNYEEWAKDLTDSQREALDGYARQDYKEINNYLRNQGGSGNEKLDAQIKNISDALGKKPIPENITVYRWCGMPEFGYQISDPLPSLKDFEEQFLNTIKEDKGYMSTSLSSERLAAFGSRKIILRLQVPKGSTGAYLSAIGGFASEKEILLDKDSKYHIDKVTEVIIKGVKRYVVDATLLTN

**Supplementary data 3:**

| **Sr. No.** | **Protein Name** | **Number of Peptide-Spectrum Matches** | **Summed Unique Peptide Precursor Intensity** | **Protein Sequence Coverage (%)** | **Summed Morpheus Score** |
| --- | --- | --- | --- | --- | --- |
| 1 | Cry 48Ab | 6 | 12938.29 | 9.18 | 37.19 |
| 2 | Cry 70Bb | 5 | 22644.52 | 6.63 | 25.21 |
| 3 | Cry 39Aa | 4 | 8222.80 | 8.49 | 26.16 |
| 4 | Cry 41Ab | 4 | 12422.02 | 6.51 | 25.09 |
| 5 | Cry 30Da/Ea | 3 | 7583.95 | 4.094 | 19.13 |
| 6 | Cry 49Ab | 3 | 6302.87 | 9.64 | 17.09 |
| 7 | Cry 57Aa | 3 | 6300.64 | 4.27 | 16.21 |
| 8 | Cry 51Aa | 2 | 7453.03 | 15.86 | 16.16 |
| 9 | Cry 35Aa | 2 | 5335.46 | 10.91 | 14.06 |
| 10 | Cry 19Ba/Ca | 2 | 15128.76 | 3.36 | 13.06 |
| 11 | Cry 53Aa | 2 | 16945.11 | 4.33 | 13.06 |
| 12 | Cry 56Aa | 2 | 9334.80 | 6.03 | 13.04 |
| 13 | Cry 33Aa | 2 | 5220.016 | 16.13 | 11.05 |
| 14 | Cry 64Aa | 2 | 6880.14 | 15.08 | 10.11 |

**Supplementary Data 3**: Proteome data of the toxin proteins identified in customized database from 9,815 MS/MS spectra of E3

**Results**

5,982 total (2,991 target + 0 decoy + 2,991 on-the-fly decoy) proteins

598,541 total (299,179 target + 299,362 decoy) non-unique peptides

9,815 MS/MS spectra

1,329 target (13 decoy) PSMs at 0.978% PSM FDR (8.069 Morpheus score threshold)

750 unique target (7 decoy) peptides at 0.933% unique peptide FDR (9.034 Morpheus score threshold)

190 target (1 decoy) protein groups at 0.526% protein group FDR (9.097 summed Morpheus score threshold)

0.63 minutes to analyze

The unique peptides identified in the proteins of S2 corresponding to larvicidal toxins are highlighted in the sequences

1. >cry48Ab gi|156712245|emb|CAJ18351.1| Crystal toxin [Lysinibacillus sphaericus]

MDINNNNEKEIINSHLLPYSLLKKYPIKSLQSTNYKDWLNLCQDFNKDIESYDLVTAVSSGTIVVGTMLS

AIYAPALIAGPIGIIGAIIISFGTLLPLLWNESENNPKTTWIEFIRMGEQLVDKTISQTVFNILESYLKD

LKVNLVDYEKAKQDWIELKKQQLPGSPPSTKLRNAADIAHQRLDSLHNKFAELNKFKVEPYETILLPVYA

QAANLHLNLLQQGAMFADQWIEDKYSSRNDTFAGNSNDYQNLLKSRTITYINHIENTYQNGLNYLWNQPE

MTWDIYNEYRTKMTITALDLMALFPFYNKELYDPTVGIKSELTREIFINTPVEPHLHRYFKLSETEEKLT

NNSDLFKWLTSLKFRTLYQPGFPFLIGNMNSFTNTNGTQLINNQQQLWSFPGTTENEEKLFPSPANIDQV

TMYIYYGSGWGIPEPISTTINKLIFNHDKHELISEYDAGNTNAPTRSLSLGLPNHYLSCLNSYYPLTATT

DGMNKEELKMYSFGWTHNSVDFLNEISKDKITQIPAVKAYRLTSNSRVIKGPSHIGGNLVYLSENSQMAL

TCRYTNSSPQEYKIRIRYASNRLNMGQLFTTFSSHQFVLPPTFNHFNIEQAKYEDYAYAEFPESMSIRGN

LNSDILLILNILAGGELLLDKIEFIPLTQKVKDNLEKEKIDMLKNLTDSLFNSPSKDTLKIDSTDYQIDQ

IAFQIESINEEINPQEKMELLDNIKYAKKLNQLRNLLYSRESQAQIDWVTSNDVSIYHGKKPFNDYTLVM

SRTSSSLSEITATNYQTYIYKKIEESKLKPYTRYLVRGFISNSEDLEIFISRYENEIHTNMNVHGDDDTL

LNSDIRQNECESKLPIIFDATSQYSLSPSRTSGISNHSYYNNGHQSSCNDTHIFSFSIDTGEVDFNNYPG

IEILFKLSNTNGYASISNLEVIEERLLTEEEKRQIIQIENRWKAKKESQRNETEKITTQAQQAINSLFTD

TQYSNLKFETTKQNITEANTILENIPYVYNALLPTEPGMNFVLFNSFKDQINKAHALYKMRNLIKNGDFI

NDTKYWSISTDVKLEKVNKETILVLSSWEAQASQQILVQKQKRYLLRVIAKKEDMGRGNVIISDCLNNIA

KIDFTPHDCNMNHIQNSSEFIIKTIHFSPNTEQVRIDIGQSDGVFKVESIELICVNY

1. >cry70Bb gi|228715456|gb|EEL67276.1| 83-kDa crystal protein [Bacillus cereus AH603]

MGTGDFMTKKQKKILSITLATGVFAGTYIPTAYTAFAENEKKEDLQENQTKNINQNSLPIDSYGWFENPY

KGVTFSQFIDAFNNNQWQPLLVNIKNKGDAGSGTISFLKGMMTTGLSLLPPPASLLASIWSVFIPTNDAN

GTDMWRQLEIYIDEKIDSKINDYHKYLMGAEFNGAMSAIKEYQRVLQIYNDSKNSLKRVEEPGTPVIEAV

RAADRKLKEFIAVVQTPEKSNDSVYQQITAPIFVQAANAHLLLQRDMILYGEEWGMDKDQWQGYKDNQKK

LIQEYTNYAMKVYNDGLEKRKKEAEEINTQQPNRNTDRWNHINDYVREYTLSVLDFVDLFPATNPETYSK

GVMQENSRQIYSSIKGAVIPQGGTGEGTTWENIQKILDSQEYKGDLHKLDIRSFDRIDAIQPWFSDKQNG

GSNWTTPGWTGNASGGALKNLINPSDNPITRVKAQSSRTPNYIDFKFDTGGDNPYFGRYFSGGSYKEDIF

EYPNQKLSQIHAFNRSTYPGFEGIDAVVFGFVDKNLTQSSTYLMTNMITTIPAAKYNRGMSNFQPQVESI

HAKQKAMKTDTTNSYLAYGVEISKEQEYKIRYKVAANENSKISLSHRKPGGNYAKIDDTTIPITGNAADT

VKGEYGSYKIVEGPIVKLTKGAHDLKLENSQGKFSLDQIELEPVERDQVIARDNFDDQRLNWINLGGIVN

GGITGKAGMIGTNGDTWTYIQDQVLPFSKYTLSIKVKLDSSDGNERQKVTIFTDNLKHERITKTVELKGKAGYQEMQLEFITSRDLANTHVGILTSNGTSNVLFDDVQVIGAKKS

1. >cry39Aa gi|19386614|dbj|BAB72016.2| mosquitocidal toxin, partial [Bacillus thuringiensis serovar aizawai]

MNSYENKNEYEILNDSKKSNMSNPYLRYPLANDSLASMQNTNYKDWLTMCDRTDTDVLSSRGAVSTGVGM

LSTILSLFGIPLIGEGIDLLLGAADFLWPESDGGHQYTWEDLMNHIEELMDERLETEKRTTALDDLRGLK

ALLGLFRDAFDSWEKNQNDPIAKNRVGGYFEDVHTHFVKDMASIFSATNYEVLLLPVYAQAANLHLLLLR

EGVIYGSRWGIAPAADFYHDQLLKYTAIYANHCVTWYNNGLAQQKELFAKSPNWNRFNAYRRDMTITVLD

IIALFPTYDARLYTKPIKTELTREIYSDVLNLDVYGVQQTDLNKNEAAFTRSPHLVTRLRGFDFYTRTKY

AYWRYLAGHTNYFSFTGNGTIYSSSFNNWYDTDMTKSTINIPDYANIYKLWTKSYTNISPYTDPVGISQM

QFSLTNNQQLTYTGTSAPKYPVRETFFEIPPTDEKPLTYENYSHILSYMTSAQHFGDKKIGYTFAWMHES

VDFDNRVDPDKITQIPAVKGDYLQYGYVKQGPGHTGGDLVSMIRTDRLGINVYFPQPLDYRIRIRYSTSS

NGYLYIYSPNTKIVYLPPTTLVDGQPTFDPMDFSAFRVVEVPASFRASVAGYTNFTIEAGFGPVYIDKIE

FIPDNTTTLEYEGGRDLEKTKNAVNDLFTN

1. >cry41Ab gi|51090236|dbj|BAD35163.1| cancer cell-killing Cry protein [Bacillus thuringiensis]

MNQSCNNNGYEVLNSGKGYCQPRYPFAQAPGSELQNMGYKEWMNMCTSGDPTVLGGGYSADVKDAVITSI

NIASYLLSVPFPPAGVAAGILGALLGLLWPTNTQAVWEAFMNTVEALINQKLDEYARSKAISELNGLKNV

LELYQDAADDWNENPGDLRNKNRVLTEFRNVNGHFENSMPSFAVRNFEVNLLPVYAEAANLHLLLLRDAV

KFGEGWGMSTDPGAERDDMYRRLRSRTEIYTDHCVNTYNQGLQQAKSLQANVSDYSRYPWTQYNQSGGFS

YREAKGEYRGTENWNLYNAFRRDMTILVLDIIAQFPTYDPGLYSRPVKSELTREVYTDIRGTTWRSDANL

NTIDAIENRMVGSRQLQLFTWLTEMKFYIRNTGSITSYTHGDLMVGLEKKIRKTNDNDQWLPLEGQNTSY

TRIDRPGIELGKNYWYYARTQQWFETRLLQLWVNTDVLSLNAGTVGNEFWARDVPDYRNIYARSTRNHFI

ENHRLSWIKFEPVRDNCPFAWPGYKQLSALLFGWTHNSVDPFNTIASDRITQIPAVKGYLVDNGATVVRG

PGNTGGDLVRLPAYNQQWTQLRVKVRPSTTARTRGYNVRIRYASEGNANLFVGKYVDTANRFYETGNYAV

NQTFSGSMTYNSFKYLDAIGFAANEEEFRIELRCNSGGPIYIDKIEFIPVNPIPEPPEGIYQIVTALNNS

SVVTSEEFCMGIGLTTRCGVNLWSNNGNTLQKWRFVYNGDQNAFQIKSTPNEDLVLSGSNSGTSVTAETN

QNRPNQHWLIEEAGNGYVYLRSKGNPNLVLDVAGTSTANGTNIILWNYNGSTNQKFKLS

1. >cry30Da gi|125661890|gb|ABN49951.1| Cry30-like protein [Bacillus thuringiensis]

MNPYQNTNECEILDDLPNYSKMVNAYSRYPLANNPEVPLQNTSYKDWLNMCQTITPLCTPIDSDINSIAA

AIGVIGSILGLIPGPGEAIGLILGTFSSIIPFLWPENKSIIWEEFTHRGLHLIRPELTPTEIEIIVTPLK

GYYNALREQLENFESEFAKWALEKSPANTRDVVLRFSAIDAAIINLKNQLIVDVRNKPAFLSLYAQTANI

DLILFQRGDKYGDEWVKYARNQPIPFKTSQEYYNSLKDKIENYTNDIAETYRNGLNIIKNIPKISWDVFN

LYRREMTLSALDLVALFLNYDIYRYPISTKTELTRKVYMSSFYLQALEQNESLESLENKPLPHSPSLFTW

LKRLNLYTISEDYSIPLRVSSLSGLSAVYSHTHQQQALYTRPYQGVLVGQPQEIRFDGFVYKLFMSQNIS

PNSCYLIGGIPQMSFYISDYSGGLRPNKDYYSASASIYFINSYMNGPQNATKSNNISIRETKHILSDIKM

NYSKTGGFYPFHTFGYSFAWTHTSVDPDNLIVPNRITQIPAVKAHYLSATAKVIAGPGHTGGDLVALIND

GSRTGSMNIECKTGSFTQPSRRFGLRMRYAENNQFSVSILRDNQGVGTFNIERTFSRTNNIIPTDLKYNE

FKYNNYDEITMDLPPNKIIDIRIQQTNSLSINQFIIDRIEFYPIDQGVEACKMQ

>cry30Ea gi|186920384|gb|ACC95445.1| Cry 30Ea1 [Bacillus thuringiensis]

MNSYQNTNEYEILDASQKNSTMSNRYPRCPLANNPQVPLQNTSYKDWLNMCQTITPLCTPVETVSDYVAA

FIGVPGSIFGAMPGPGAAVGLFLSSFSTIIPILWPNDTTPIWKEFTKQGLQLFRPELGRDAIEIIGNDVQ

AEYNALKTMMQDFETKFATWDLNRTRANAIAATTEFNSVKNQIIRLQERFLIAAENRPAFLNLYAQTANI

DLILYQRGAANGDKWLEDINNRSISPFSSKDYYQDLKLKIKNYTNYCAETYRNSLNILKNKSDIQWSIYN

GYRRVATLGALDLVALFPNYDICIYPIQTQTELTRKVYMPSFYSERLPKGNIETWENSLTHPPSLFTWLK

KLDPYTKSERFNPALEVASLCGLHAALSYTPQNGPEFAGPFQGILGTKTTPLISFDNQFVYELFLTQYRH

PNDCYSISGKPKITFYISDYYGNSRPNKEYSSNIQLSSVITSYMNGPQNASTSNNISIKQTKHILSDIKM

IYTQIGGIYPSHDFGYSFAWTHTSVDPDNLIVPNRITQIPAVKAYSLTSPARVIAGPGHTGGDLVALLNN

NLEAGRMQIQCKTGSFTGASRRYGLRMRYAANSQFTVNLSYVLSGTTYGTSFITESTFSRLNNIIPTDLK

YEEFKYKEYSQIITMTLPANTIITISIQQAVASSNYQLIIDRIELYPMDQDVVACTVN

1. >Cry49Ab gi|156711540|emb|CAJ86542.1| Cry49Aa protein [Lysinibacillus sphaericus]

MENQIKEEFNKNNHGIPSDCSCIKEGDDYNSLTEVPSEINAKEFSYCSPNMFNLNLPEQSTRFQTIGSIH

SNNCSFEILNNDPGYIYGDSVAGECRIAVAHRELGNGLERTGDDRFLFIFYALDNNNFIIANRHDGFVLQ

FLIANGQGVIVSREYQPNIRQEFTIQSINSDTFRLHSRDTNTFATVCWAQFNSWTKIVSRVDNPGAPNAD

LRHRSFLDINMPQLPSLTPLQPLPRLTGLEDGGLSPAQAPRAIIGRTLIPCLFVNDPVLRLESRIKQSPY

YVLEHRQYWHRLWTDIFNAGERREYREVTGINHNAQNDMNNMINITIGSDGPNRLLFGHLSTPFRQQIIS

NSNTLGSFANSNYSSRTESITYLNTEFHQVRFARFVKAYEYRLTRADGTLVGTPWVVLDRKEMDLRTFPH

NMTLNLENVKIVNADNSYDLSVWKTPLKLKDGKIIIENHENSKPYYN

1. >cry57Aa gi|225348555|gb|ACN87261.1| Cry delta-endotoxin [Bacillus thuringiensis serovar kim]

MGTWWPTDSASDTWGEMIGFAQELVGTALSEDLKIRTNQQIDSIRIALQAYYSSLEDWLNANKPLSGPLL

NQVTEEFGNALRKSRDSIAYFKSDDSNVYTIILLPAYAQVANFHLALIHEGLKYATEWNLPRLQTFGYEE

DLKHYTISYVNHCEYWYQKGLDILYPRNVIGMTQWMKRNFYRLNMTINVLDIISLFSLYDSKKYPNFFED

IYNAQKELISKFQLTRIVTTEPTLHQHKYLNDSSKKICQNESSCDPIDLDEYLTLPLMFQNWLRNINFQY

LAPVISVGDEALYPFFVATQNINEYMNAEGNMIIGRQQGLWNFQFIIVHLFHLVYKKMTIFMVKWLSAYP

LIDEDILNGVRTPYILQKIEFYNLNKSINSKQIRPISAGTTKSPLIDIYYGLPDVNGYNLDEPQFNFNAA

SHYFNSIQTCYYKENTSKNHYDIYQSYVFHWEHASVKRKDEVVSDRITIFPAIKSNPILSRGIQIISHQG

HTGGNVIYFTPQSELHFKINFVSNRQKYKIRLRYVAFNPVVIQYHGSNSYASLSSITLPRTSSNQNVRDL

RYEEFGYSDFEINMSSAGGLEDIKIISNNEFILDRIEFIPDTLFNYLSN

1. >cry51Aa gi|112253719|gb|ABI14444.1| Cry51Aa1 [Bacillus thuringiensis F14-1]

MIFLAILDLKSLVLNAINYWGPKNNNGIQGGDFGYPISEKQIDTSIITSTHPRLIPHDLTIPQNLETIFT

TTQVLTNNTDLQQSQTVSFAKKTTTTTSTSTTNGWTEGGKISDTLEEKVSVSIPFIGEGGGKNSTTIEAN

FAHNSSTTTFQQASTDIEWNISQPVLVPPRKQVVATLVIMGGNFTIPMDLMTTIDSTEHYSGYPILTWIS

SPDNSYNGPFMSWYFANWPNLPSGFGPLNSDNTVTYTGSVVSQVSAGVYATVRFDQYDIHNLRTIEKTWY

ARHATLHNGKKISINNVTEMAPTSPIKTN

1. >cry35Aa gi|16554960|gb|AAG50342.1| 43.8 kDa insecticidal crystal protein [Bacillus thuringiensis]

MLDTNKVYEISNLANGLYTSTYLSLDDSGVSLMSKKDEDIDDYNLKWFLFPIDNNQYIITSYGANNCKVW

NVKNDKINVSTYSSTNSVQKWQIKAKDSSYIIQSDNGKVLTAGVGQSLGIVRLTDEFPENSNQQWNLTPV

QTIQLPQKPKIDEKLKDHPEYSETGNINPKTTPQLMGWTLVPCIMVNDSKIDKNTQIKTTPYYIFKKYKY

WNLAKGSNVSLLPHQKRSYDYEWGTEKNQKTTIINTVGLQINIDSGMKFEVPEVGGGTEDIKTQLTEELK

VEYSTETKIMTKYQEHSEIDNPTNQPMNSIGLLIYTSLELYRYNGTEIKIMDIETSDHDTYTLTSYPNHK

EALLLLTNHSYEEVEEITKIPKHTLIKLKKHYFKK

1. >cry19Ba gi|3426160|dbj|BAA32397.1| insecticidal protein [Bacillus thuringiensis]

MNSYQNKNEYEILDAKRNTCHMSNCYPKYPLANDPQMYLRNTHYKDWINMCEEASYASSGPSQLFKVGGSIVAKILGMIPEVGPLLSWMVSLFWPTIEEKNTVWEDMIKYVANLLKQELTNDTLNRATSNLSGLNESLNIYNRALAAWKQNKNNFASGELIRSYINDLHILFTRDIQSDFSLGGYETVLLPSYASAANLHLLLLRDVAIYGKELGYPSTDVEFYYNEQKYYTEKYSNYCVNTYKSGLESKKQIGWSDFNRYRREMTLSVLDIVALFPLYDTGLYPSKDGKIHVKAELTREIYSDVINDHVYGLMVPYISFEHAESLYTRRPHAFTWLKGFRFVTNSINSWTFLSGGENRYFLTHGEGTIYNGPFLGQDTEYGGTSSYIDISNNSSIYNLWTKNYEWIYPWTDPVNITKINFSITDNSNSSESIYGAERMNKPTVRTDFNFLLNRAGNGPTTYNDYNHILSYMLINGETFGQKRHGYSFAFTHSSVDRYNTIVPDKIVQIPAVKTNLVGANIIKGPGHTGGDLLKLEYERFLSLRIKLIASMTFRIRIRYASNISGQMMINIGYQNPTYFNIIPTTSRDYTELKFEDFQLVDTSYIYSGGPSISSNTLWLDNFSNGPVIIDKIEFIPLGITLNQAQGYDTYDQNANGMYHQNYSNSGYNYNQEYNTYYQSYNN

>cry19Ca gi|391224596|gb|AFM37572.1| crystal protein [Bacillus thuringiensis serovar vazensis]

MDSYHNKNEDEMLNASLNQSNMDNRYPLANYPNKSLQNTNYKDWLTMCEGTPVVFASEAQAFKVLGATIA

RVLGLIPAVGPLLSSLVSIFWPTLQTPNTIWQDMMKYVADLIRQELTTYTINQATRNLTGLYESLNIYNR

ALAAWKINKNHFASAELVRGYINDLHIRFGADIQADFTLKGYETILLPSYASAANLHLLLLRDISVYGKE

LGYSQQDLDFYYGEQKHYTERYSNHCVNKYNAGLNLEKQKGWSSFNRYRRDMTLLVLDLVALFPLYDLRI

YPSKDDNINVKSELTREIYSDVINAHVYLVLNEDMAYFAQAEALYTRQPHLFTWLRGFRFVTNSISSWTF

LSGSQNKYSYTNNNSIFNGPFYGQDTEYGGTSSNMDIAEGSYIYQLWTKNYEYIYPWLDPVNITKINFSV

TDNNFSKEVTYGGERINIPTVRTDFDFLIKKDGTGLATHNNYSHILSSILTNGSTAGQKKHGYSFAFTHS

SVDQKNSLSFDKITQIPAVKSSDWLFYGNLLKGPGHTGGDLVFLDNGNNFNVRVNFPVQSYRVRIRYAAD

GNGEMAISVDGTLYTPFNVERTFSNNNYNDLKFEDFKVIDTPLIYNASYEGAKSIFLYNNSNKRVIIDKI

EFIPIGKSALEYESKQNLEQAQKAVNDLFTNDTKNMLKTDTTDYQIDQVVNWVDCVSEELYVKEKMILRD

EIKYAKQQSLSRNLLQNGDFEDTSKGWTTSNTITIQADNPIFKGHYLNMSGAREIDGTIFPTYIYQKIDE

SKLKPYTRYQVRGFVGSSKGLEFVVTRYGKETDAIMNVPNDWPYIQPNSSCGDYHRCDTSSEPVMYQGYP

TPLPEGYAPDLGLLCQNSLGKKHVVCHDRHQFDFHITTGELDINTNLGIQVLFKISSPDGYATLNNLEVI

EEGPLSGESLERVKHREKKWKQNMEAKRLETQQAYNAAKQVVDSLFTNAKDESLRFDTTLTHIMNAEHWV

QSIPYVDNAWSSDIPGTSNDLYVELEARLAQARYLYDAQNVITNGNFTQGLMGWHATRDVEVQQMNGASV

LVLSNWSAGASQNVHAQHHQGYVLRVIARKEGTGKGYVTMMDCNNNQETLTFTSCEEGYITKTVEVFPDT

DSVRIEIGETEGSFYIESIELNCMKGYYDQKSDSIYDQGYNNNYNQNSSNMHNQGYKNNYNQNGSSAKFL

KR

1. >Cry53Aa gi|157418804|gb|ABV55105.1| Cry toxin [Bacillus thuringiensis]

MNSYQNKNEYEILDASQNNSTMSNHYPRYPLAKDPLASMQNTNYKDWLNLCDTPNMENPEFQSVGRSALS

ILINLSSKILSLLGIPFAAQIGQLWSYTLNLLWPVANNATQWDIFMRTIEDLINVRIETSVRNRALAELE

GLGNILEDYKVALRRWDLNPTNLDRQSEVVSQFEIVHAFFRVQMPVFAIRDFEVPLLPVYASAANLHLLL

LRDVVINGDRWGLSAARINDYHDLQLRLTSTYTDHCVNWYNTGLNRLIGTNARQWVTYNQFRREMTISVL

DIISLFSNYDARRYPTKTQSELTRMIYTDPIGAVGTIGLNPGWLDNAPSFSVIENSVVQSPRTFLFLERV

GIFTGVLHGWSSQSQFWSAHRLFLSNLSSIWESIIYGNPQNNIGYEEVDFTNFDVFSINSRATSIMSPFG

GGELFGVPRVTFDLSNRTNNSLAQRTYNRPFTFGGQDIVSRLPGETTEIPNSSNFSHRLAYISSFRVGIA

GSVLSYGWTHHSVDRHMRLNPNMITQIPAVKXVSGHIVSGPGHTGGDILRVHSGSQGTIIIQSNSAQRYR

LRLRYSSTLPGDLILNHRGTDGSQQFIEFTLPATSGQLRFADFTYADGRTVFQTPNSHVFYTVHVQTRSN

GIFFIDKIDYIPENTPPLECGGERNLEKEKKAVNDLFTN

1. >cry56Aa gi|256033941|gb|ACU57499.1| pesticidal crystal protein [Bacillus thuringiensis]

MNSYQNKNEYEISDASRNNSNMSTRYPRYPLANNPQMPMRNTNYKEWLAMCADNKDNEQVPISPLDQEWR

TVLPILFSAAASLTGLISLPTFGAISAGAAIAAALTSILFPSQGPDVFNELMGATENLLKREIETYVRGR

AASELLALEDQRAYFKSAFDYWRLHPTDGNAIATVAARFHTVNGAFVTAMRLFRPAGYEALLLPVYAQAA

RLHLLHLRDGVLFANEWGLAKPDPGDLHDQEFNAKAAEYADYCELTYNTELNRIKTTSGKTWFDYNQYRR

LMTFAVLDVVAKFSILNPRIYSLALQGEILTRKIYTDPVNFSPGSSIADDENRYTVPPSHVRQLVNSRLF

TNVASVQNAGFIGNQNRYKNIGVSDLVDGPIIGQSVFEKVDADIPTNVSVFEVGVNGIQNDYPRNIGLKK

TPSNAFTNNYAGSTNNLGPFTTVSLPPKDDKPLDHTNFSHRLSDIILPGNKGSSFAWTHVDVDPTGNYLS

TTKINLIPATKASKLPLSFQLRKGPGFIGGDLVRLGNGVGISYKFNFQSSDSSANFRIRIRYAGAGSGAS

GGGQVYFKLGNYQSPDTSWGHTGFDSNNVKYNQFKVLELLGTAGNITANDLEIIVWTRDPGASDFYLDRL

ELIPMTGISTEYNEPQKLETAKKAVNDLFTNNL

1. >cry33Aa gi|16588691|gb|AAL26871.1|AF316145_2 crystal protein NT40KD [Bacillus thuringiensis serovar dakota]

MAIIDFNQKIKDFCQWQVSNEFNPGRDLRNWVISYENTNISAVPRGASGGVATVNVTPVLKVASTQELYN

GSSVTQSQTARFSETTQETQSSTTTEGARFSSTVTSTTKFTANVNFKAIGSGIDQTIAVAVTGEYNYSSS

QTITTQRTRVWDVTQPVIVPPRTRIVATLLIYDAPFSIPIDLNCEVTGKIPINNGFSQDLAGATYDYTIG

TNTRASSYTRLGMMAYYNWPGKVPEFVGYQSAGRAAFATLVYRGVGLQTAVQGLYSVVKFEEFPIGRQGE

TRTYYSPIQLADQNTSLAPDSNTIPIINPM

1. >cry64Aa gi|294661779|dbj|BAJ05397.1| crystal protein [Bacillus thuringiensis]

MAIFDVEADLIDNNKWYAQKYYNANPSTFRNPIVYDMNVSDLDVVPITTEFSSTPQLTNSATQVVRNNTS

KDQSQTVLFSEKSIETFSRSTTEGYKIGSSIKSTTSFKVKVGFLVSGEINQSIEVAITGEYNHSSTETTT

TTNEKLWQVTQPVIIPPYTQVTATLQIFSGPFVVPAKTKATIQGKGTNNGAYNFASAITYTDNSGRVYTD

RNRAQALYTDRNEWPGYKRIYVGGSSSTDPTGLLRLEGEARITAQVGLYAVTEFRESPLPGYAGVGSNRT

YYAPNILLGDGSVIQFPEYQRYLQR

**Supplemantary Data 4:**

| **Sr. No.** | **Protein Name** | **Number of Peptide-Spectrum Matches** | **Summed Unique Peptide Precursor Intensity** | **Protein Sequence Coverage (%)** | **Summed Morpheus Score** |
| --- | --- | --- | --- | --- | --- |
| 1 | Cry 56Aa | 9 | 37393.70 | 23.83 | 56.23 |
| 2 | Cry 25Aa | 7 | 36803.77 | 12.89 | 45.18 |
| 3 | Cry 33Aa | 7 | 26386.62 | 44.19 | 44.13 |
| 4 | Cry 32Aa/Ba/Da | 6 | 27914.58 | 3.20 | 23.15 |
| 5 | Cry 52Ba | 5 | 24776.13 | 7.68 | 32.17 |
| 6 | Cry 49Ab | 4 | 11457.30 | 12.21 | 26.16 |
| 7 | Cry 30Da/Ea | 4 | 10393.42 | 5.70 | 22.11 |
| 8 | Cry 24Ba/Ca | 4 | 6274.86 | 3.64 | 15.07 |
| 9 | Cry 47Aa | 3 | 14991.91 | 4.14 | 20.16 |
| 10 | Bin B | 3 | 13804.71 | 17.19 | 20.06 |
| 11 | Cry 51Aa | 3 | 12050.69 | 18.12 | 18.1 |
| 12 | Cry 45Aa | 3 | 9938.67 | 9.82 | 16.1 |
| 13 | Cry 42Aa | 2 | 6253.92 | 5.19 | 17.05 |
| 14 | Cry 39Aa | 2 | 18361.42 | 3.94 | 16.10 |
| 15 | Cry 9Bb | 2 | 7871.13 | 3.78 | 16.03 |
| 16 | Cry 54Aa | 2 | 18964.89 | 4.61 | 15.08 |
| 17 | Cry 38Aa | 2 | 6379.75 | 10.32 | 14.07 |
| 18 | Cry 63Aa | 2 | 12559.32 | 5.31 | 14.05 |
| 19 | Cry 14Aa | 2 | 14789.87 | 2.87 | 14.05 |
| 20 | Mtx-2 | 2 | 7156.43 | 16.78 | 13.06 |

**Supplementary Data 4**: Proteome data of the toxin proteins identified in customized database from 10,741 MS/MS spectra of S3.

**RESULTS**

5,982 total (2,991 target + 0 decoy + 2,991 on-the-fly decoy) proteins

598,541 total (299,179 target + 299,362 decoy) non-unique peptides

10,741 MS/MS spectra

409 target (4 decoy) PSMs at 0.977% PSM FDR (9.225 Morpheus score threshold)

272 unique target (2 decoy) peptides at 0.735% unique peptide FDR (10.047 Morpheus score threshold)

86 target (0 decoy) protein groups at 0.000% protein group FDR (11.065 summed Morpheus score threshold)

0.80 minutes to analyze

The unique peptides identified in the proteins of S3 corresponding to larvicidal toxins are highlighted in the sequences

1. >cry56Aa gi|256033941|gb|ACU57499.1| pesticidal crystal protein [Bacillus thuringiensis]

MNSYQNKNEYEISDASRNNSNMSTRYPRYPLANNPQMPMRNTNYKEWLAMCADNKDNEQVPISPLDQEWR

TVLPILFSAAASLTGLISLPTFGAISAGAAIAAALTSILFPSQGPDVFNELMGATENLLKREIETYVRGR

AASELLALEDQRAYFKSAFDYWRLHPTDGNAIATVAARFHTVNGAFVTAMRLFRPAGYEALLLPVYAQAA

RLHLLHLRDGVLFANEWGLAKPDPGDLHDQEFNAKAAEYADYCELTYNTELNRIKTTSGKTWFDYNQYRR

LMTFAVLDVVAKFSILNPRIYSLALQGEILTRKIYTDPVNFSPGSSIADDENRYTVPPSHVRQLVNSRLF

TNVASVQNAGFIGNQNRYKNIGVSDLVDGPIIGQSVFEKVDADIPTNVSVFEVGVNGIQNDYPRNIGLKK

TPSNAFTNNYAGSTNNLGPFTTVSLPPKDDKPLDHTNFSHRLSDIILPGNKGSSFAWTHVDVDPTGNYLS

TTKINLIPATKASKLPLSFQLRKGPGFIGGDLVRLGNGVGISYKFNFQSSDSSANFRIRIRYAGAGSGAS

GGGQVYFKLGNYQSPDTSWGHTGFDSNNVKYNQFKVLELLGTAGNITANDLEIIVWTRDPGASDFYLDRL

ELIPMTGISTEYNEPQKLETAKKAVNDLFTNNL

1. >Cry25Aa gi|3668335|gb|AAC61892.1| insecticidal protein Jeg74 [Bacillus thuringiensis serovar jegathesan]

MNPYQNKSECEILNAPLNNINMPNRYPFANDPNAVMKNGNYKDWLNECDGITPSIFGTLGVLASIVISTI
NLATSPSIGDAFALVSSIGEYWPETKTSFPLSVADVNRLIREALDQNAINRATGKFNGLMDTYNTVYLKN
LQDWYDTRIPANPQGDSQLREAARRSLEEIERDFRKALAGEFAEAGSQIVLLPIYAQAANIHLLILKDAM
QFRTDLGLIRPVGVPITTSAEDPFESEFLLRIKKYTDHCISYYDDGLAKIRSRGSDGETWWEFNKFRREM
TLTVLDLVALYPTHNIKLYPIPTQTELSRVVYTDPVGCFGNRKSDIFSRLNFDYLENRLTRPREPFNYLN
SVQLFASTVSNSNNGEVLRGNLNKIMFEGGWTASRSGDGVTTGTPFSTMDWSYGWGYPRKHYAEITSRSQ
ALPGLNNSIHVIVGIDSFRAIGPGGQGDHTFSLPGGDMYDCGKVQINPLEDYRNSDHWISDMMTINQSVQ
LASNPTQTFAFSALSLGWHHSSAGNRNVYVYDKITQIPATKTVREHPMIKGPGFTGGDLADLSSNSDILQ
YDLRSDYDDRLTEDVPFRIRIRCASIGVSTISVDNWGSSSPQVTVASTAASLDTLKYESFQYVSIPGNYY
FDSAPRIRLLRQPGRLLVDRIEIIPVNFFPLSEQENKSVDSLFIN

1. >cry33Aa gi|16588691|gb|AAL26871.1|AF316145_2 crystal protein NT40KD [Bacillus thuringiensis serovar dakota]

MAIIDFNQKIKDFCQWQVSNEFNPGRDLRNWVISYENTNISAVPRGASGGVATVNVTPVLKVASTQELYN

GSSVTQSQTARFSETTQETQSSTTTEGARFSSTVTSTTKFTANVNFKAIGSGIDQTIAVAVTGEYNYSSS

QTITTQRTRVWDVTQPVIVPPRTRIVATLLIYDAPFSIPIDLNCEVTGKIPINNGFSQDLAGATYDYTIG

TNTRASSYTRLGMMAYYNWPGKVPEFVGYQSAGRAAFATLVYRGVGLQTAVQGLYSVVKFEEFPIGRQGE

TRTYYSPIQLADQNTSLAPDSNTIPIINPM

1. >cry32Aa gi|15721993|gb|AAG36711.1| crystal protein [Bacillus thuringiensis serovar yunnanensis]

MDPNDHNEYEVMDTGSMGYQPRYPLANAPGAELQQMHYKDWMDMCTYGESVALSEGLRAALFVANSIIGI

MLSKIPIVGPIVSTPFQIMGVALPFLWPPNAPEPQFSWESLMTAAEEIADKKIDAQVRANANAELEGVHN

AIRLYQDAVCDWKQDPTNAQLKEQLRIQYIATNTVIFSRMPSFRVRGFEVPLLSTYVQAANLHLIHLKDG

VQFGEEWGMDSATVDRFYSYLKSDIEIYTNYCIDWYNKGLSDSIESEPTWNGWNTFNNFRRDMTLMVLDL

VSIWPTYDPRRYPLPTKSQLTRELYTQAIGSYKSVEPLLPPPSPFRWLREIEFFLRDSQDEAEQFAGFQQ

GYQYTLDTTIYRPPVVGTRTSLVDSIAMGLGSDDVVYRIKNISHNGWYPKKLDFYYTPSERVESVGEIRT

DANNMIDYIGLGCRAKVTEPCDPCTTNCTIDTVNTTASCDNPNLYSHRLSSINPSAPYPGQNGMLSFCYG

WTHFSVDDNNLIAADSITQIPAVKAYRIGGYGKVMKGPGYTGGDLMVFYGAGEINWRLTIPDTTKAYRVR

ARVATIPTSQPVDTVRYNQFEYRDIEVISPTTTVHEYSIGFDITGDDWGLLDKIEFIPIEGPVEAYQADQ

ALEKARKAVNALFTNDAKNALQLKVTDYSVDQAANLIECVSDAFHSQEKMILLDQIKYAKRLSQARNLLN

YGDFESSDWSGENGWRTSPHVHVASNHPIFKGRYLHMPGATSSQFSNHIYPTYVYQKVDESKLKSYTRYL

VRGFVGNSKDLELLVERYGKDVHVEMDVPNDIRYALPTNECGSLDRCRPASYQARTPHTCTCKDTTSMHT

DCQCQNKVNRTSADMYTNGSPSSVMYADGFHAHKSCGCKNNDMYPNGTDSHKSCRCKDPHVFSYHIDTGC

VDQEESLGLWFALKIASENGVANIDNLEIIEAQPLTGEALARVKKREQKWKQEMTKKRLETEKAVQAAQS

AIQNLFTNAQHNRLKFETLFPQIVHAEKLVEQIPYVHHPFLSGALPTVPGMNFEIIQQLLAVIGNARALY

EQRNLVRNGTFSSGTGSWKVTEGVKVQPLQDTSVLVLSEWIHEASQQLHIDPNRGYVLRVTARKEGGGKG

TVTMSDCADYTETLTFTSCDFNTSGSQTMTSGTLSGFVTKTLEIFPDTDRIRIDIGETEGTFQVESVELI

CMEQMEEDLYDIAGNVVEEMRYLDSSRSMGGTLDAMCYTKIGEFGC

>cry32Ba gi|17385646|dbj|BAB78601.1| crystal protein CryE6L [Bacillus thuringiensis]

MNQNDNKNEYEILDSGNLSYQPRYPLANAPGSKLQNMGYKEWRDMCPDAKRQLLKQIGISPFEPDEWTKV

GQSLTISAGLVAAIAGVASVAFPPLAIVAGAFAIISMFFDVLWPESENNTNSQVVWADFAAAAEEMMDEK

IADEIKAEAVLQLRIVQSRLRDYQQAACNFQSDPNNESYKALLRDAFDDADDALKEVMILFSREGYEQLL

LFDYVQAANLHLLLLKDVVRFGVGWGFPPGRVEQYYSNPTNLGNPGMVQLLAKYTNYSTSLCWKGIEERK

WMVESEYRSNNEEYYAYRSNMTMMVLDMVALWPTYDPVKYPYATAVELTREIYSLIAGGFRDYKGYMPIQ

YTWEKPGSLVTNLERFTIYTWKNFEYFPFAGVETTYQTIGTGSSSTKQAGVIPSPEEGTAWTTPGINVEY

HLVESFIYGTLTQLLFYDVYNTPAFKAGSDAVEVPGSIAGTPCKNIPLDANDVNVCVPTLWEESPANPEG

VCYPYNREMQSNLLSEVIPEDPKLLTLPHVPPLGYIDAFAFAWRSTTCDTRYNLIPANKIGQIPAVKGNW

LGQSGSSVVRTSGNTGGDVVRLYEFGNLGMTVRFSENRSYIIRLRYATVADDLNIIVRVQRRGELEYESE

LPLNQTSNNSTTQWKFEDYGYQEVGGFYPQVGEEYELWFSPVGTELSSHMDIDKIEFIPMETSLEEYLAN

QDIEKARKAVNALFTGDVKNALKLNVTDYAIDQAANLVECVSEEFYAQEKMILLDQVKVAKRLSQARNLL

NYGDFESPEWSRENGWKTSRHVSVRADNPVFKGRYLHMPGVTSPSFSNNTYPTYVYQKVDESKLKSYTRY

LVRGFVGNSKDLELLVERYGKDVHVEMDVPHDIRYSLQTNECGGFDRCRPVSYLARSSHACTCKDTASMH

TDCQCQNKANRTVTNMYTNVSPGSAMYTDGFHAQKSCGCKNNDMYQNGTHPHKSCGCKDPHVFSYHIDTG

CVDPEENLGLWFALKIAGENGVANIDNLEIIEAQPLTGEALARVKKREQRWKQERDKKRLETEKAVQTAQ

GAIQNLFTNTQQNLLKFETLFPQIVNAEMLVQQIPYVYHPFLSGALPAVAGMNFKIVQQLSATIGNARSL

YNQRNLVQNGTFSSGTGSWHVSEGVEVQPLQNTSVLVLSEWSHEASQQVSVDPDRGYVLRVTARKEGVGK

GTVTLSDCADYTETLTFTSCDFNTSGSQTMTSGTLSGFVTKTLEIFPDTDRIRIDIGETEGTFKIESVEL

ICMEQMEDD

>cry32Da gi|17385650|dbj|BAB78603.1| crystal protein CryE6S [Bacillus thuringiensis]

MKQNYNDEYDIIDNGGRGDQSRYPLTNAPSAELQDMNYIEWLDNCTLKEQVELFRDTSTTVRDALATTAG

IITALLSVSNPAAAATAGIITILIPQLWPSGSDEVTWEKFMAAAEILIQKQITEAVRNKALTELEGVYRT

IRLYQLAAERWNQNKNDPQAQESIRTQFRATNTIIEFAMPSFRVAGFEVPLLNVYAEAANLQLALLRDAV

KFGRDWGLPQDEVDDIYSEQLLPRIAEHTDHCVTYFNRGLEEAKKLKANLNDYARYPWAQYINHSKIQGI

ENWNLFNDYRRNMTILVLDLVALWPTYDPRRYSMVTKSELTRELYTSVRGAFYGHNNDYDQNFEEIERNI

ISPPHLVTWPINFTVYTQNDYYYPMAGLQHKFNYTESIVSLESPVTGVTGTSNLINFVTADPFILAVTII

GFGQLGTSLGIYAMSFGRKSGSISHVGDIEIGTDDYLDIVDRIPVGDSTPNKLSWMSAAPTTLGSTTFLQ

YVSYAWRHPSVDSNNRISIDKITQIPAVKAFFIDDNHVKVIKGPGSTGGDLVAFSREGYGLSISVFIPGS

DLVSFYRVRIRYASSQSAKVTMGFGGGVEESESKFNFPATYSGGNLTYNSFGYINTLAIASQSTAQTIEV

YFRQYEEAEFIVDKLEFIPMEMSLEEYQADQDLEKARKAVNALFTSDAKSELKVNITDYAVDQAANLVEC

VSEDFHAQEKRILLDQVKFAKRLSQARNLLNYGDFESLDWSGENGWRTSPHVHVASDNPIFKGRYLHMPG

AMSPQYSNNTYPTYAYQKVDESKLKSYTRYLVRGFVGNSKDLELLVERYGKEVHVEMDVPNDIRSTLPMN

ECGGFERCGKVSYQAVSDHTCTCKDTARMNTDCQCKDKINHMTTGVYTSMPVGSAMYPDGYHAHKSCKCG

DKTMYGNGKHPHKSCGCKDPHVFSYHIDTGCVDQEENLGLWFALKIASENGVANIDNLEIIEALPLTGEA

LARVKKREHKWKQEMEQKRCKTEEAVQAAQTAINTLFTNTQYNRLKFETLFPHILHADELVQRIPYVYHP

FLLGAYPDVPGMNYDIFQQLSALVNQARGLYDMRNLVRNGTFSAGIGNWQVTDGVATQPEGNTSVLILRE

WSDKAIQHLRIHAERGYVLRVTARKEGNGDGYVVIHDCDNQQEKLTFTACDYTTMGSSTGTQTMMTSPTN

CMPCNSTTWKEEMKALVPMLSGYVTKTAEIFPDTDRIHIEIGETEGTFKIESVELICMEHMEEHAYDMEG

DIEANIPPIVRPPIMPPTNV

1. >cry52Ba gi|227976386|gb|ACP43735.1| pesticidal crystal protein, partial [Bacillus thuringiensis]

MNSYQNKNEYEILDASQNNSNMSNRYPRYPLANDPQASMQNTNYKDWLATCNGTPAPLYNSSQLLKISGN

VVSRALGMLPIPGIAPLLSFLSTLLWPSGSSGNTIWESLMKEAADLIDQKLEENILRQATANLAGLQGLL

GSYNSAFASWEAGGNATPDLVKGYMESLHRTFVQDIIGSFTIPGYEKILLPTYAITANFHLMLLRDIEIY

GGKKTPEGKDGLNFDPKDLNFYNCELKKYKELYTNHCLNTYNKGLASEKEKGWVPFHRYRREMTLAVLDI

IALFPLYDARLYPAKNNKEMPVKSELTREIYSDVINSDRFGVVPPYNYAQNEERYTRPPHLFTWLRGLDF

VTNVLTSGTWVYRWSVLTGLSKEIFLYKREWYYNWSFSGLSCRVWWKNFQHYYCRRFLYLELVAKKLSIY

FPLVFYDKYRTDYFLTNKNNSSTEKVYGYVAGNANLPTVQTDFDFLTNKEVTGPPTYNNYNHILSYLLLG

YDWNQTGGIGTHGYSFAFTHSSVDPYNTIAPDKITQIPAVKAFEISDAGPSQVIAGPGHTGGDVVRLYLS

GRLKIRLTPASTNKNYLVRVRYASPVSGTLRVERWSPSSVTNRDFTRLATGGFNSFGYVDTLVTTCNQSG

VEIIIQNLGASDVIIDKVEFIPYDIPIDKCTKCEFEGNVCTCRCEGVQSLEKEKEIVNSLFVKENKVCTE

VGG

1. >Cry49Ab gi|156711540|emb|CAJ86542.1| Cry49Aa protein [Lysinibacillus sphaericus]

MENQIKEEFNKNNHGIPSDCSCIKEGDDYNSLTEVPSEINAKEFSYCSPNMFNLNLPEQSTRFQTIGSIH

SNNCSFEILNNDPGYIYGDSVAGECRIAVAHRELGNGLERTGDDRFLFIFYALDNNNFIIANRHDGFVLQ

FLIANGQGVIVSREYQPNIRQEFTIQSINSDTFRLHSRDTNTFATVCWAQFNSWTKIVSRVDNPGAPNAD

LRHRSFLDINMPQLPSLTPLQPLPRLTGLEDGGLSPAQAPRAIIGRTLIPCLFVNDPVLRLESRIKQSPY

YVLEHRQYWHRLWTDIFNAGERREYREVTGINHNAQNDMNNMINITIGSDGPNRLLFGHLSTPFRQQIIS

NSNTLGSFANSNYSSRTESITYLNTEFHQVRFARFVKAYEYRLTRADGTLVGTPWVVLDRKEMDLRTFPH

NMTLNLENVKIVNADNSYDLSVWKTPLKLKDGKIIIENHENSKPYYN

1. >cry30Da gi|125661890|gb|ABN49951.1| Cry30-like protein [Bacillus thuringiensis]

MNPYQNTNECEILDDLPNYSKMVNAYSRYPLANNPEVPLQNTSYKDWLNMCQTITPLCTPIDSDINSIAA

AIGVIGSILGLIPGPGEAIGLILGTFSSIIPFLWPENKSIIWEEFTHRGLHLIRPELTPTEIEIIVTPLK

GYYNALREQLENFESEFAKWALEKSPANTRDVVLRFSAIDAAIINLKNQLIVDVRNKPAFLSLYAQTANI

DLILFQRGDKYGDEWVKYARNQPIPFKTSQEYYNSLKDKIENYTNDIAETYRNGLNIIKNIPKISWDVFN

LYRREMTLSALDLVALFLNYDIYRYPISTKTELTRKVYMSSFYLQALEQNESLESLENKPLPHSPSLFTW

LKRLNLYTISEDYSIPLRVSSLSGLSAVYSHTHQQQALYTRPYQGVLVGQPQEIRFDGFVYKLFMSQNIS

PNSCYLIGGIPQMSFYISDYSGGLRPNKDYYSASASIYFINSYMNGPQNATKSNNISIRETKHILSDIKM

NYSKTGGFYPFHTFGYSFAWTHTSVDPDNLIVPNRITQIPAVKAHYLSATAKVIAGPGHTGGDLVALIND

GSRTGSMNIECKTGSFTQPSRRFGLRMRYAENNQFSVSILRDNQGVGTFNIERTFSRTNNIIPTDLKYNE

FKYNNYDEITMDLPPNKIIDIRIQQTNSLSINQFIIDRIEFYPIDQGVEACKMQ

>cry30Ea gi|186920384|gb|ACC95445.1| Cry 30Ea1 [Bacillus thuringiensis]

MNSYQNTNEYEILDASQKNSTMSNRYPRCPLANNPQVPLQNTSYKDWLNMCQTITPLCTPVETVSDYVAA

FIGVPGSIFGAMPGPGAAVGLFLSSFSTIIPILWPNDTTPIWKEFTKQGLQLFRPELGRDAIEIIGNDVQ

AEYNALKTMMQDFETKFATWDLNRTRANAIAATTEFNSVKNQIIRLQERFLIAAENRPAFLNLYAQTANI

DLILYQRGAANGDKWLEDINNRSISPFSSKDYYQDLKLKIKNYTNYCAETYRNSLNILKNKSDIQWSIYN

GYRRVATLGALDLVALFPNYDICIYPIQTQTELTRKVYMPSFYSERLPKGNIETWENSLTHPPSLFTWLK

KLDPYTKSERFNPALEVASLCGLHAALSYTPQNGPEFAGPFQGILGTKTTPLISFDNQFVYELFLTQYRH

PNDCYSISGKPKITFYISDYYGNSRPNKEYSSNIQLSSVITSYMNGPQNASTSNNISIKQTKHILSDIKM

IYTQIGGIYPSHDFGYSFAWTHTSVDPDNLIVPNRITQIPAVKAYSLTSPARVIAGPGHTGGDLVALLNN

NLEAGRMQIQCKTGSFTGASRRYGLRMRYAANSQFTVNLSYVLSGTTYGTSFITESTFSRLNNIIPTDLK

YEEFKYKEYSQIITMTLPANTIITISIQQAVASSNYQLIIDRIELYPMDQDVVACTVN

1. >Cry24Ba gi|50539655|dbj|BAD32657.1| delta-endotoxin [Bacillus thuringiensis]

MVNPYQNKKESVFYEHSNNQKDIPNRYPFTNNPNAVMKNGNYKDWVNECEGSNVSPSPAAAVTSAIISIV

LKTLAKALVSSLVDAIKSSLGISEVITKNNVSQLSMELVNQLINRRIQETIMDLGSASLNGLMSIYKRYL

NALEAWDKDKSNITLQENVIEEFKYVESRFFENLKGIYRTSSSQITLLPTFAQAANLHLSMLRDAVMYQE

GWNLQSHLDYKMELDIALKDYTNYCVEVYNRGLNALRGSTALDWLEFNSFRRDMTLMVLDLVAIFPNYDP

VQYPLPTKIGLSRKIYTDPVGTTRDTDFGNWTLTDRTLANFNDLERDVTDSPSLVKWLVDMNIYTGAIDS

YPISGPGGERIGVWYGNMNSFVLTGSRELSYNMYGEIAHEDPITNIRDNDIYKVDLRAAYVATIRNALDS

TFGVSSSHFFNVMGKNELYQSKQPYPSYPITITFPGEESLEGNVNDYSHLLCNVKNITGGLRQTSARGRS

SLLSHAWTHKSLNPKNIIAADKITHIPAVKGSNLSASSAVIKGPGFTGGDLLRLGPNQFVDYILTPDNPQ

VSQIYFDVRLRYACMGGANILIQFWNKNWEIGVQLVSTTSSLENLKYENFAYITTRLSFTFGQGGYNMSI

YNPTSNPNVIIDKIEFIPVSGTPFEYEGKHKLKNTQADVNNLFLN

>cry24Ca gi|134274695|emb|CAJ43600.1| pesticidal crystal protein cry24-like [Bacillus thuringiensis]

MNPYQNKNKYEILESSSDYTKISNKYPFANDPNITMKHMNYKDWMHMCETPTPFSASIIISVISIFGRVL

GLSSSFANTSKYIAIITGILGLLGSSSSGSTNVWDNLIKHIEELTDRFIDTSVRFTGMSTVNGLNSQYLY

YLDLFEDWYEDQNNEQKRNRLVGTFQTLGLAFINALGYRNTNGQEVRGTLSTAYEVQFLPSYAQAANLHL

LLLKDAVTYGDKWILIEPTSNYYNRFKDNIARYTDYCTEYFHKGLDNLKKPGSDAVSWLRFNGFRRDMTL

MVLDLITLFPLYDSVQYPLPTQIELSRQIYTDPVGATFSDFSNWTLTNRTLANFNDLEREVTDAPSLLKW

LNSIEVYTGAIDSHRPTSPGERIGVWYGNRNTYINPDTNEVSYRFTGELAYENPYTFIGSFFDNDIYKVA

LRAAAVSTTFGSNDSTFGVSSSQFFNRRGIHQLYESSQPIPSWPITLAFPGEESSEGNANDYSHRLCDVK

NITGGLRQTPARGRSSLLSHAWTHSSLKLRNIIAADKITQIPAVKTWEIRGTSSVVAGPGNTGGNLVKMS

YHSVWNIKFTCQQLKRYRVRIRYASDGNCQLAMRRWRGGPGYVQEARHTVQRTFSGSMTYDSFKYLDIFT

MPAEDYTFNLTIDLESGGALYIDKIEFTPDDITTLEYEGERDLEKTKNTVNDLFTN

1. >cry47Aa gi|62997562|gb|AAY24695.1| Cry [Bacillus thuringiensis]

MNSNDSQNHDKLEICSNASLLSTPPYKTEYYEGEVLMDTIPFSSGKSAAQMGTSIVGQILGVLGVPFALQ

VTNLYSSLLDTLWPDGKSQWEIFMEQVEEIVDQKIENYARNKALAELEGLGNNFEVYLEALENWQSNTRD

INDVKIRFISLDSLFTQSMPSFRIEGFQLPLLSVYAQAANLHLLLLRDATTFGKEWGLDSATMDSYYKRQ

KALSAEYSDHCVKWYKNGLNKLSKSTAKDWVKFNQFRREMTLTVLDVVALFPNYDAKIYPMQTVTQLTRE

VYTDPVGMTNLPNGIGSWYDIAPTFATIENAVIRKPHLFDFIRNLYVYFGDRGASANRSMKFWNGHEIFY

ANIGSSVFFQVYNYVKLGSNNYPYVFVDRDIYQTESLAGCIYDIIYPGYTYKFFGAPKVEFHVVDRRNNT

GIFTFNPGFGQIIQNVQNSLSQLPLESLDEPAYEAYSHRLCHVTLVPPGNNSNYEGLPVYSWTHKSASLE

NVIYPDKITQIPAVKSFPGGQWGGVEAGPGFTGGDVTKSVTSEATTILRDVVKLAVTIPQDSIKQKYRVR

IRYASQTDIPATFFTSDSGNRDFVLKSTTTAASNAFENYKEFQYIDIPGTIEFTKVNEIVTVYLHAYKVS

NHHVNIDKVEFIPVDNNFEARKQLETSEFFAKKLLDTTKESLDREVTDYQIDYTAKLVECVSDELYPMEK

QELLNIVTTAKKLSQDRNLLQDIDFSAINRENGWIGSRGIEGTEGNINFKSRSVRLPGARNIDGKIDSTY

FYQKINASKLKPYTRYELRGRIESSKKLEIYLIHHNANRIIKNVNGNNSLLNSYNEIDPCISKCSNHILS

IEEAELDMNNHTNFHEFSLCIDTGELDLNKNIGIWVAFKISDLNGYAELRNIECIEVEPLFGEALEKLKK

QEQEWTRTENKQYEESKKIRAVAIKAVEQLFEDSSYQKLRPELDLSNISKAENLVNSIPYVSNDWFSYVP

GINHNTVEELKTKIQLAFALYRHRNSIQNGDFKNGLNSWTVTSDVVVEENQIDPELVISNWSSQVSQDVV

VEANHRYLLRVTAKKENVGIGYITIHDSTDNSKSIVFDDCEKNQDHYVTKTTEFIPSTDKVYIKIHETDG

IFRIKNIDFTLNKK

1. >BinB gi|166034391|gb|ABY78896.1| **binary toxin B [Lysinibacillus sphaericus]**

MCDSKDNSGVSEKCGKKFTNYPLNTTPTSLNYNLPEISKKFYNLKNKYSRNGYGLSKTEFPSSIENCPSN

EYSIMYDNKDPRFLIRFLLDDGRYIIADRDDGEVFDEAHTYLDNNNHPIISRHYTGEERQKFEQVGSGDY

ITGEQFFQFYTQNKTRVLSNCRALDSRTILLSTAKIFPIYPPASETQLTAFVNSSFYAAAIPQLPQTSLL

ENIPEPTSLDDSGVLPKDAVRAVKGSALLPCIIVHDPNLNNSDKMKFNTYYLLEYKEYWHQLWPQIIPAH

QTVKIQERTGISEVVQNSMIEDLNMYIGADFGMLFYFRSSGFKEQITRGLNRPLSQTTTQLGERVEEMEY

YNSNDLDVRYVKYALAREFTLKRVNGEIVKNWVAVDYRLAGIQSYPNAPITNPLTLTKHTIIRCENSYDG

HIFKTPLIFKNGEVIVKTNEELIPKINQ

1. >cry51Aa gi|112253719|gb|ABI14444.1| Cry51Aa1 [Bacillus thuringiensis F14-1]

MIFLAILDLKSLVLNAINYWGPKNNNGIQGGDFGYPISEKQIDTSIITSTHPRLIPHDLTIPQNLETIFT

TTQVLTNNTDLQQSQTVSFAKKTTTTTSTSTTNGWTEGGKISDTLEEKVSVSIPFIGEGGGKNSTTIEAN

FAHNSSTTTFQQASTDIEWNISQPVLVPPRKQVVATLVIMGGNFTIPMDLMTTIDSTEHYSGYPILTWIS

SPDNSYNGPFMSWYFANWPNLPSGFGPLNSDNTVTYTGSVVSQVSAGVYATVRFDQYDIHNLRTIEKTWY

ARHATLHNGKKISINNVTEMAPTSPIKTN

1. >cry45Aa gi|48290384|dbj|BAD22577.1| parasporin 1470D [Bacillus thuringiensis serovar shandongiensis]

MAIINLANELAIWAKRWCAARGYTYLVSGLQAHTGNYGRIYNYNMSVPDPIVTDNPTNAAMARGTTPNPT

SQPIIRTISFNETLTDSQSTATEHGITAGAEVTVKSEAGLIFAKVGFEVKVSFQYNYTTTNTYTTETSRS

WTDSLQITVPPGYVTEHTFIVQTGPYSKNVVLEADIAGHGWFNYSAPGYTGTGIVNITQVLYDNKVPGVT

PYPDNFYARFRGSGKLEGKMGLQSFVNLVERPLLGRAGQVREYQIPVSLPSGLDIPIFDPVVSLQ

1. >cry42Aa gi|51090240|dbj|BAD35166.1| Cry protein [Bacillus thuringiensis]

MNQNYNNNGLEILDSGGVCSPRYPLANAPGSELQNMGYKEWLEMCSIKGAETFADKSTLSAQSQEGLRTA

ITIALSLLSNLPGPFGYPAKLLSIIFPFLWPTNTQAQWEAFMKVVEELVDQKIETFARDQAIQRLRGIQD

VISLYQRDAKNFNDYPTSEPIQRQLLSQFTATNTFIVGSMSLFRVGRHEVPLLTTFVQAANLHLLLLRDA

IMFGESWGMCPVTVAGYQNDFNNRIADYTDYSVSIYNQGLQKAKTLKANLRDYEKYPWARYYNSSVGPEF

AYGDMENWNLYNNYRRDMTLMVLDLVALWPTYNPQQYPIAPKIQLTREIYTELRGNAGNTKRPSMDAIDA

ELIPPPRLFTWLESVDMHRWPTSAGYYYYTFQNAGIKHRYKYTLDSQTLTSSLRGASGNNFNLVPAEETI

NRVQNQHGEGLYTFSFYRSGQSDPFLNIGTTADKPYVSTMNRIPVEGDQTQANHRLSWITGMVIPELSIP

AFGHYNPTYISCAAEGWTHLSVERSNEIKSDKITQIPAVKAFQLSNNASVVRGPGSTGGDLVQFSATSSG

NKQLWIKVKPTTIALGRRFKVRIRYAAAANVTFTVQKCVTGVACWETATKSVTTTYSGTLTYNAFKYVDI

FEIPANESEFSLEFLSTSGGPIYIDKIEFIPVNPIPEPPVPEGIYQIVTALNNSSVVDMDPGTWGTRHNV

HLWQNNNTNNQKWRFVYNSSQGAYQIRNLADENLVLTREGANVKVVSYQNNNTAQYWIIEDAGNEYVYLK

SKADPSRVLDVTGSSTQNGTNIQVWSNYGTLNQKFKLVKL

1. >cry39Aa gi|19386614|dbj|BAB72016.2| mosquitocidal toxin, partial [Bacillus thuringiensis serovar aizawai]

MNSYENKNEYEILNDSKKSNMSNPYLRYPLANDSLASMQNTNYKDWLTMCDRTDTDVLSSRGAVSTGVGM

LSTILSLFGIPLIGEGIDLLLGAADFLWPESDGGHQYTWEDLMNHIEELMDERLETEKRTTALDDLRGLK

ALLGLFRDAFDSWEKNQNDPIAKNRVGGYFEDVHTHFVKDMASIFSATNYEVLLLPVYAQAANLHLLLLR

EGVIYGSRWGIAPAADFYHDQLLKYTAIYANHCVTWYNNGLAQQKELFAKSPNWNRFNAYRRDMTITVLD

IIALFPTYDARLYTKPIKTELTREIYSDVLNLDVYGVQQTDLNKNEAAFTRSPHLVTRLRGFDFYTRTKY

AYWRYLAGHTNYFSFTGNGTIYSSSFNNWYDTDMTKSTINIPDYANIYKLWTKSYTNISPYTDPVGISQM

QFSLTNNQQLTYTGTSAPKYPVRETFFEIPPTDEKPLTYENYSHILSYMTSAQHFGDKKIGYTFAWMHES

VDFDNRVDPDKITQIPAVKGDYLQYGYVKQGPGHTGGDLVSMIRTDRLGINVYFPQPLDYRIRIRYSTSS

NGYLYIYSPNTKIVYLPPTTLVDGQPTFDPMDFSAFRVVEVPASFRASVAGYTNFTIEAGFGPVYIDKIE

FIPDNTTTLEYEGGRDLEKTKNAVNDLFTN

1. >cry9Bb gi|54112021|gb|AAV28716.1| Cry9Bb delta-endotoxin [Bacillus thuringiensis serovar japonensis]

MNRNNQNEYEVIDTSTCGCPSGDVMQYPLANDPNAALQNMNYKEYLQMYGGDYADAFINPGNVRTGLQTG

IDIVALLVGTLGGAVGGILTGLLSTLFGFLWPSNDQAVWEAFIEQMEELIEQRISDQVVRTALDNLTGIQ

NYYNQYLLAFEEWEEAPNSVRSNLVLQRFENLHALFVSSMPSFGSGPGSQRFEAQLLLVYAQAANLHLLL

LRDAEIYGARWGLRESQIQLYFDELQNNTRDYTNHCVNAYNNGLEQVRGTNAASWLKYHQFRRETTLTAM

DLVALFPYYNLRQYPIAVNPQLTREVYTDPLGVPFEESNPSSEIRCSRWQDTSAMTFSNLENALVRPPHL

FDTIRNLRIYTGTFRVNNNNFIEGWIGHSVTNNRLGISTEFTRNYGITTPIINSYNFANGDVYQINTRSN

TSLIAFENAPLFGITRAQFQPGGTYSVTQRTLLCEQNYNSTDELPSLDPDEPISRSYSHRLSHITSYLHR

VFTIDGNNIYSGNLPTYVWTHRDVDLTNTITADRITHLPLIKSNVQRSGLPVKGPGFTGGDVLRSSSSDA

DVSIIGVSAGAPLTQQYRIRVRYASNVDVTIRFVRHNTHSLLGIGTLSRTMNSGEESRYESYRTVDVITN

FRLNSSSEQIRIVTEGLRANGQLYLDSLEFIPINPTREAEEDLEAAKKAVASLFTRTRDGLQANVTDYQV

DRAANLVLCLSDEQYAHDKKMLLEAVRAAKRLSRERNLLQDPDFNEINSTEDSGWKTSNGIIISEGGPFF

KGRALQLASARENYPTYIYQKVDSSMLKPYTRYKLDGFVQSSQDLEIELIHHHKVHLVKNVPDNLVLDTY

PDGSCNGINRCEEQQMVNSQLETEHHPMDCCEASQTHEFSSYIHTGDLNASVDQGIWVVLKIRTTDGSAT

LGNLELVEVGPLSGESLEREQRDNAKWNAELGRKRAEADRVYQGAKQAINHLFVDYQDQQLNPEVGLAEI

SEARNLIESISDVYCDAVLRIPGINYEMYTELSNRLQQAAYLYTSRNAVQNGDFNSGLDSWNATTDATVQ

QDGNMYFLVLSHWDAQVSQQFRVQPNCKYVLRVTAKKVGNGDGYVTIQDGAHHRETLTFNACDYDVNGTH

VNDNSYITKELEFYPKTEHMWVEVSETEGTFYIDSIELIETQE

1. >cry54Aa gi|169261091|gb|ACA52194.1| insecticidal crystal protein Cry54Aa [Bacillus thuringiensis]

MSMKSLIQRIYLVLLTEILTILDTLLQIIRINHYKNWINMCQKNQQYGENLETFASADTIAGVSAGVIVV

GTMLGAFAAPITAGLIISFGTLLPIFWKPGEDPKTVWQAFLKIGNRPFSSPVDQALIDLLSNKARSLESQ

FNDFQRYFDIWNNNKTPGNAGEVLRRFSSLDADIIRELEQLKGNYYITVLPGYAQVANWHLNLLRIAAFY

YDQWASSSNLSIQSIYPEDYINDLQTCLTNCAIESGNKISSKYYKCVLKCRINEYINYCSKTYQEGLNIL

KNSSGLKWNEYNTYRREMTLNVLDLIAVFPNYDPDKYLISTKSQLTREIYTDALIDAFANAHFNINDIEN

SLTRPPGLVTWINRLDFYTGMFTKSVPGLTANGINYSFTNGNSNDSPIYGYRLSDDSSTPIQIPRNQYVY

NMLITYLRDSPSVIQKIEFNLNNQQTRTYDTGLTLAPTYQSTINLSLPGKDRSFPPKFNNYTHFLSYVKT

APGDERPSSSRARNVCFGWMHFSVNDYDVLAGGYNTISNKIITQIPAVKARHLPLPSFVMPGPGHTGGNL

VVLSTQIEFQCIVLNPVSYKIRMRYVAYSPNRSINLTVSIRSEIGNYQNIVPNISSTVQSPEDTKNPKYE

HFQYLDISIPLELFGITNITITRSDSISNNTLIIDKIEFTPDV

1. >cry38Aa gi|14571533|gb|AAK64559.1| crystal protein ET75 [Bacillus thuringiensis]

MSILNLQDLSQKYMTAALNKINPKKVGTFHFEEPIVLSESSTPTRSEIDAPLNVMFHASQDLDNRRGTSD

LKQTVSFSQTQINTVETKTTDGVKTTKEHTFSGTLELKIKYAMFDLGGVSGTYQYKKSTENDISSEKSKS

KSDSQTWSISSEYTVKPGVKETLDFYIVGIKTEVPLNIFAEFQGTKTIDNVSNVMAYQEFISQDDEHIRA

CMKASKLANPDHLSGYTAPKELKANTSKGSVEFRGTAIAK INTGVKCLVVVNGKNSITGKTYSYIHPKTM

LADGTIEYLESEIDLLESEIDLLTTSSILV

1. >cry63Aa gi|260268375|dbj|BAI44028.1| M019CP84 [Bacillus thuringiensis]

MSVVYYVKGGDIMDPFSNCSEKKYSDSNNNQELTVESSSFYSNTTNENMKKNYPPINKNFSRNSNDTVLD

ILNISQNNNIDIFAPYNNLHSIKDELQIRTVIPGGVIFSVTGNKFVDSKFTAITYAAITKLTSSLITAAA

TAILGPIGTTIGGAISGPIANALFGLIPGMKPLTPQEIIDIAVEQSKLYTDEQITNLVITNATSELASIK

AKIEDFNSQLNFALNSKNDNLMNRIDFETFLVTLENDIYGSIIKLMNFGYSKQLLPIITVCCTLNLSFLR

DAIFNSQTFNISTQGKRVLTDTFERRTIEYSDKIINEYTLLFNEIKLKENAKTTLDFRTFMSLQVLDQVD

LWSVFKFSQFNIRNTRRLYTIPYQYSENDVSKLDPTQINGDWKFINQILYGLPGNRISGFAGTVELYQPN

KIRRINKLKALYSNNETTGYVGKDANAMDSFDTHPIISQKPAIINYASHVIVNLPPSSVQNLTLLDTIGP

IFPGKYVINQQLYPGLNSLLEYEKFAIPDHKGVNVAGLPNIDSSYTSSTIDNLRQNFITSKPILGSVTAF

QKDIPNYEQVNNKEQIVHLCPTDTDQKLLGFNIPALEYSKDRIANFGFEETWMIIPSYSSSGDNLQFKGT

TAGIKYYLKSAQNAYANYKIFIKIAYKPNNSGNKVQLNINMK.DLTSSSIISATLNIQNTSLLKGTSEDNVKFITFEVPTNFPISNNTYELQLIFTNLQQNDDLRLNELILHPINNDFINILNA

1. >cry14Aa gi|538378|gb|AAA21516.1| delta endotoxin [Bacillus thuringiensis]

MDCNLQSQQNIPYNVLAIPVSNVNALVDTAGDLKKAWEEFQKTGSFSLTALQQGFSASQGGAFNYLTLLQSGISLAGSFVPGGTFVAPIVNMVIGWLWPHKNKTADTENLIKLIDEEIQKQLNKALLDQDRNNWTSFLESIFDTSATVSNAIIDAQWSGTVDTTNRQQKTPTTSDYLNVVGKFDSADSSIITNENQIMNGNFDVAAAPYFVIGATLRLSLYQSYIKFCNSWIDAVGFSTNDANTQKANLARTKLTMRTTINEYTQRVMKVFKDSKNMPTIGTNKFSVDAYNVYVKGMTLNVLDMVAIWSSLYPNDYTSQTAIEQTRVTFSNMVGQEEGTDGTLKIYNTFDSLSYQHSLIPNNNVNLISYYTDELQNLELAVYTPKGGSGYAYPYGFILNYANSNYKYGDNDPTGKPLNKQDGPIQQINAATQNSKYLDGETINGIGASLPGYCTTGCSATEQPFSCTSTANSYKASCNPSDTNQKINALYAFTQTNVKGSTGKLGVLASLVPYDLNPKNVFGELDSDTNNVILKGIPAEKGYFPNNARPTVVKEWINGASAVPFYSGNTLFMTATNLTATQYKIRIRYANPNSDTQIGVLITQNGSQISNSNLTLYSTTDSSMSSNLPQNVYVTGENGNYTLLDLYSTTNVLSTGDITLKLTGGNQKIFIDRIEFIPTMPVPAPTNNTNNNNGDNGNNNPPHHGCAIAGTQQLCSGPPKFEQVSDLEKITTQVYMLFKSSSYEELALKVSSYQINQVALKVMALSDEKFCEEKRLLRKLVNKANQLLEARNLLVGGNFETTQNWVLGTNAYINYDSFLFNGNYLSLQPASGFFTSYAYQKIDESTLKPYTRYKVSGFIGQSNQVELIISRYGKEIDKILNVPYAGPLPITADASITCCAPEIDQCDGGQSDSHFFNYSIDVGALHPELNPGIEIGLKIVQSNGYITISNLEIIEERPLTEMEIQAVNRKDQKWKREKLLECASVSELLQPIINQIDSLFKDANWYNDILPHVTYQTLKNIIVPDLPKLKHWFIDHLPGEYHEIEQKMKEALKHAFTQLDEKNLIHNGHFATNLIDWQVEGDARMKVLENNALALQLSNWDSSVSQSIDILEFDEDKAYKLRVYAQGSGTIQFGNCEDEAIQFNTNSFVYKEKIIYFDTPSINLHIQSEGSEFVVSSIDLVELSDDE

1. >Mtx2 gi|1378030|gb|AAC44120.1| **Mtx2 [Lysinibacillus sphaericus]**

MKRTKLLFYIMIASFLFVNGSIYTAKATTIDENNHDIIKQQGVSIEDIDRKIDNMIASIPPLFGFLPYSR

FPYIFGESVDVSGINIENTNVTSVVPLFIGSNTFENTTDRTMTFNTVSFSKSITDSTTTQTLNGFKTAFE

ASGKVGIPLVAEGQIKTTLEYNFSHTNSNTKSVTTTYTVPPQPIPVPPHTKTRTDVYLNQVSISGNVEIY

ADAITGIKAESSGTVISIGDGLNLASNTFGLIRSPQDPDRVRAIGSGKFNLIHGADFTAITYDITSGEAS

ARIIDVKEISFK

**Supplementary data 5:** Data of the metabolites identified for the three *Enterococcus* species using LCMS. A cutoff of 99.5% was used to search for the spectral peaks against the standard databases for the available with the instrument. Metabolites common to all four or at least three of the *Enterococcus* species among strain S1, S2 and S3 are highlighted in various colors.


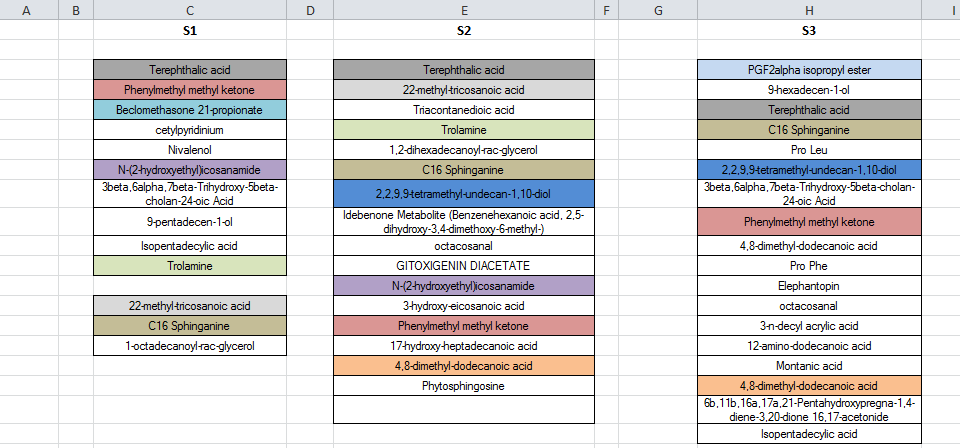

Supplement: Supplementary file 1 — Supplementary data. [file 41598_2020_61245_MOESM1_ESM.docx]
